# Intelligence without intuition: a mixed-methods pilot study on reasoning models in musculoskeletal physiotherapy for low-back pain

**DOI:** 10.3389/fdgth.2026.1789412

**Published:** 2026-03-18

**Authors:** Ricardo Knauer, Matthias Kalmring, Erik Rodner

**Affiliations:** 1 KI-Werkstatt, University of Applied Sciences Berlin, Berlin, Germany; 2 Institute of Health Sciences, University of Applied Sciences St. Pölten, St. Pölten, Austria

**Keywords:** clinical reasoning, large language models, mixed methods, musculoskeletal, reasoning models

## Abstract

Musculoskeletal pain, especially low-back pain, is highly prevalent and often challenging to manage due to its multifactorial nature. Effective diagnosis and therapy require clinicians to integrate biopsychosocial information within an evidence-based clinical reasoning framework. Large language models that “think” before responding, so-called reasoning models, show promise to support such complex decision-making, yet their validity and reliability in this setting remain unclear. In our work, we present a comprehensive human evaluation of reasoning models for clinical reasoning. Our results indicate that state-of-the art reasoning models demonstrate sufficient test–retest reliability and are competent or proficient in terms of their conceptual reasoning, completeness, correctness, relevance, and usefulness, with no statistically significant or clinically relevant differences between them. However, our qualitative analysis reveals weaknesses in logical coherence, patient-centeredness, empathy, and intuition, with most deviations from expert reasoning in the domain of intuition. Our findings underscore the importance of adopting a multidimensional framework for evaluating language model outputs and allow us to provide guidance for model selection and prompting strategies to enhance clinical reasoning performance.

## 1 Introduction

“We are what we repeatedly do” (1). Our everyday behaviors, such as a sedentary lifestyle, shape our health trajectories and can contribute not only to the emergence of lifestyle-related health problems, but also to their long-term functional and socioeconomic burden. The prevalence of musculoskeletal disorders, for instance, is projected to more than double by 2050 (2). Low-back pain, in particular, remains the leading cause of years lived with disability and carries major societal and economic consequences (2, 3). Identifying modifiable determinants of pain and disability is therefore critical in clinical practice (4). However, musculoskeletal pain is inherently multidimensional and modulated not only by lifestyle factors, but also by physical, cognitive, emotional, and further biological and psychosocial factors (5–7). This complexity often leads to ambiguous clinical presentations and substantial diagnostic uncertainty. This uncertainty may contribute to suboptimal treatment choices and, ultimately, poorer patient outcomes as well as increased societal costs.

To determine the primary drivers of an individual's pain and disability, clinicians must engage in a systematic, biopsychosocial clinical reasoning process (8–10). Patient-centered reasoning is essential to accurately stratify patients according to their individual risk factors (9), plan targeted treatments (11), and deliver (cost-)effective care (12). Yet comprehensive clinical reasoning in musculoskeletal diagnosis and therapy is difficult (13). Evidence suggests that physiotherapists, for instance, struggle to evaluate psychosocial factors, primarily due to inadequate educational structures (14–16). Beyond the complexity of the patient presentation itself, clinicians must also integrate the best available research evidence, patient preferences, and clinical expertise (17), which further adds to the demands of clinical reasoning. Given the time constraints and growing patient numbers, consistently applying multidimensional clinical reasoning in daily practice is challenging. Innovative approaches to support clinicians in such complex diagnostic and therapeutic decision-making are therefore warranted.

In response to these demands, recent technological developments have explored digital approaches to support complex clinical reasoning. Artificial intelligence (AI) technologies, particularly large language models (LLMs), offer new opportunities to assist clinicians in managing the cognitive complexity of clinical reasoning by synthesizing diverse clinical and biomedical knowledge sources (18–21). Through web-scale pretraining on general-purpose and domain-specific text corpora, LLMs acquire the ability to generate human-like responses to a wide range of textual inputs, including queries related to diagnostic hypothesis generation, personalized treatment planning, and clinical reasoning tasks (22). LLMs that generate answers through multi-step chain-of-thought deliberation, so-called reasoning models, may be particularly relevant in clinical contexts characterized by multidimensionality, ambiguity, and uncertainty (23–25), as their stepwise processing resembles the structured integration of heterogeneous information central to clinical reasoning. Despite these advantages, the web-scale pretraining of LLMs may also introduce biases and factual inaccuracies into their responses, and the underlying model architecture limits transparency in how conclusions are derived (26). In light of these limitations and the limited evidence regarding the validity and reliability of state-of-the-art reasoning models as clinical reasoners (27), a thorough evaluation of these models in the clinical setting is warranted. In our work, we address this gap by providing a comparative analysis of reasoning models for clinical reasoning in musculoskeletal physiotherapy for low-back pain.

Our contributions are as follows:

- **We conduct a comprehensive human evaluation of reasoning models for clinical reasoning**, involving five musculoskeletal case vignettes, three clinical reasoning tasks, three state-of-the-art reasoning models, and five human experts (Sec. 2). We use a mixed-methods design to provide a multidimensional assessment across five quantitative dimensions (conceptual reasoning, completeness, correctness, relevance, and usefulness) as well as three qualitative dimensions (strengths, weaknesses, and differences to expert reasoning). Additionally, we estimate the test-retest reliability to assess the consistency of the model outputs.
- Our results suggest that the **models are sufficiently reliable and competent or proficient in their clinical reasoning**, with no statistically significant or clinically relevant differences between them across the evaluated dimensions (Sec. 3.1). However, we find that the models show **weaknesses in logical coherence, patient-centeredness, empathy, and intuition, with most deviations from expert reasoning in the domain of intuition** (Sec. 3.2). These findings motivate the need for a multidimensional approach to evaluating large language model outputs and enable us to offer **guidance for model selection and prompting strategies** to improve clinical reasoning performance (Sec. 4.4).

## 2 Materials and methods

To comprehensively evaluate the validity and reliability of reasoning models for clinical reasoning tasks within a musculoskeletal context, we adopted a mixed-methods approach (28) with a convergent design (29). Five representative case vignettes from musculoskeletal practice were selected (Sec. 2.1) and three clinical reasoning tasks were defined (Sec. 2.2). We applied three state-of-the-art reasoning models (Sec. 2.3) and invited five musculoskeletal experts to evaluate the model outputs (Sec. 2.4). This allowed us to capture both quantitative and qualitative performance measures (Sec. 2.5), enabling us to contextualize the quantitative findings (30, 31).

### 2.1 Case vignettes

Low-back pain is highly prevalent worldwide (2, 3) and shaped by a complex interplay of biopsychosocial factors (32, 33), requiring a broad spectrum of clinical competencies for effective diagnosis and therapy (34, 35). To reflect the diversity of presentations encountered in routine practice, we selected a spectrum of low-back pain cases in which the primary pain drivers were biological, psychosocial, or a combination thereof (36–39). This selection was guided by the first authors' clinical expertise and aimed to illustrate typical diagnostic and therapeutic complexities inherent in daily practice. We also included a rare case involving a sinister pathology that necessitates careful screening and urgent triage (40). All cases were drawn from peer-reviewed journals, ensuring that no sensitive information was used. The five cases are briefly summarized as follows:

1. This case describes a patient with chronic non-specific low-back pain accompanied by psychosocial risk factors (yellow flags). In particular, the patient exhibits maladaptive illness beliefs and pain-related fear, associated with fear-avoidance behaviors (36).
2. This case presents a patient with low-back pain secondary to a neoplastic malignant compression of the cauda equina. The clinical picture includes multiple warning signs of serious pathology that warrant medical referral (red flags), such as saddle anesthesia, urinary incontinence, and unexplained weight loss (40).
3. This case involves a patient with chronic non-specific low-back pain characterized by limited lumbar range of motion, reduced local loading capacity, decreased core strength, and increased paraspinal muscle tone and tenderness (37).
4. This case reports a patient with similar findings to those in case 3. Additional features include palpable trigger points in the quadratus lumborum and a high level of kinesiophobia (38).
5. This case outlines a patient with acute low-back pain following a work injury. The clinical presentation includes signs of sciatic nerve sensitization and sacroiliac joint involvement, and yellow flags such as fear avoidance (39).

### 2.2 Reasoning tasks

We operationalized clinical reasoning in three free-text tasks for the reasoning models:

1. Initial diagnostic process: From the patient history, generate a working hypothesis and choose and justify diagnostic tests.
2. Final diagnostic process: Incorporating additional diagnostic test results, generate a working hypothesis.
3. Therapeutic process: Based on the updated working hypothesis, generate a treatment plan and choose and justify therapeutic interventions.

Each reasoning model was role-prompted to act as an “expert physiotherapist with proficient clinical reasoning skills” and instructed to “summarize the reasoning step-by-step”, as the reasoning traces were not accessible for all models (25).

### 2.3 Reasoning models

We selected the top three reasoning models from the Chatbot Arena Leaderboard as of July 1st, 2025 (41):

1. Gemini 2.5 Pro: This model is a closed-source model released by Google in June 2025 (42). We set the temperature parameter to zero instead of the default value of one to reduce stochastic variation across responses, given the study's focus on systematic evaluation rather than creative generation. This resulted in outputs that were often, but not always, identical. Gemini 2.5 Pro was queried on July 4, 2025.
2. o3: This model is a closed-source model released by OpenAI in April 2025 (43). Since o3 does not support non-default temperatures, we left the temperature at the default setting of one. It was queried on July 2, 2025.
3. DeepSeek-R1-0528: Unlike Gemini 2.5 Pro and o3, this model is an open-source model that was released by DeepSeek-AI in May 2025 (44). As the temperature is ignored during inference for DeepSeek-R1 (45), we left the temperature at the default setting of one. DeepSeek-R1 was queried on July 3, 2025.

Notably, these models perform similar to or better than publicly available specialist models like MedGemma 27B or OpenBioLLM 70B (46), echoing results from earlier studies on general-purpose vs. domain-specific models (47).

### 2.4 Model output raters

The reasoning model outputs were rated by five Austrian physiotherapists. Within their professional scope of practice, Austrian physiotherapists routinely screen for red flags, perform assessments, formulate physiotherapeutic diagnoses, plan and implement treatments, and refer patients back for further medical evaluation when appropriate (48). We recruited physiotherapy lecturers with more than five years of experience in musculoskeletal rehabilitation. Participation was voluntary and anonymized. All raters were employed as lecturers at the University of Applied Sciences St. Pölten, Austria, and held a master's degree in musculoskeletal rehabilitation or a related field such as sports physiotherapy. In addition, they were concurrently working as practitioners in musculoskeletal rehabilitation in private practice. For an overview of the raters' years of clinical experience and professional specialization, please refer to Supplementary Table 2. To preserve rater anonymity, demographic information is not reported. Written informed consent regarding the study procedures and data processing was provided. As no patient data or information from vulnerable persons were collected, no formal ethics approval was required.

### 2.5 Outcome measures

The model outputs were rated by the musculoskeletal experts based on both quantitative and qualitative performance measures. The quantitative evaluation was categorized into primary and secondary levels.

**Primary outcome measure.** Our primary outcome measure was the conceptual reasoning, as described in the Clinical Reasoning Assessment Tool (49, 50). Conceptual reasoning encompasses a range of cognitive and metacognitive skills, such as formulating a working hypothesis, planning a treatment, and selecting and justifying diagnostic tests or therapeutic interventions. We employed a numeric rating scale (NRS) instead of a visual analog scale, given their demonstrated good agreement (51, 52) and the NRS' advantages in terms of greater rater acceptability, simpler scoring, and reduced measurement errors (53, 54). The ratings were operationalized from “beginner” (0–2) and “intermediate” (3–5) to “competent” (6–8) and “proficient” (9–10) (49, 50).

**Secondary outcome measures.** For secondary outcome measures, the raters used NRS' to evaluate the correctness, completeness, relevance, and usefulness of the model outputs according to their professional judgement (55–57). Additionally, we assessed both the interrater reliability and the test-retest reliability. For interrater reliability, we estimated the intraclass correlation coefficient [ICC(3,5)] among our fixed set of model output raters based on the average NRS scores (58). For test-retest reliability, we prompted each reasoning model with five identical inputs for each case vignette and reasoning task, then calculated the semantic similarity between the model outputs using gte-base-en-v1.5, a state-of-the-art long-context text encoder (59). Because embeddings with an angle <45° are more aligned (similar) than orthogonal (dissimilar), we considered models with cosine similarities >cos(45°) = 0.70 to be sufficiently reliable (60). However, text embeddings can be anisotropic, meaning that even semantically dissimilar sentences may have a high cosine similarity (61). We therefore also included a toy example to verify that our embedding model produced reasonable text representations.

**Qualitative analysis.** To identify the strengths, weaknesses, and characteristic “thought structures” of the reasoning models in contrast to human clinical reasoning, the raters were additionally asked to answer the following three open-ended questions:

1. What was particularly helpful about the generated output?
2. Where do you see weaknesses in the reasoning process?
3. How do the generated reasoning steps differ from your own?

Based on the responses, we conducted a structured qualitative content analysis (62). The main categories were deductively derived from the three questions, while the subcategories were inductively developed from the response material. Finally, the statements were systematically coded. The qualitative analysis allowed us to contextualize the quantitative results in the sense of a methodological triangulation (63).

### 2.6 Data collection

The case vignettes were identified via hand search in peer-reviewed journals (Sec. 2.1). We paraphrased the case material, segmented it by reasoning task, and removed information that could cause information leakage between tasks (Sec. 2.2). The revised case vignettes were then provided to the reasoning models (Sec. 2.3) and sent in an initial e-mail to the model output raters (Sec. 2.4). In a second e-mail, the raters received the model outputs and instructions for rating them (Supplementary Material). There was a three-day interval between the two aforementioned e-mails. The data collection period spanned six weeks from July to September 2025. The quantitative data was transferred to a Microsoft Excel sheet and the qualitative data to MAXQDA for statistical analysis.

### 2.7 Statistical analysis

We used JASP 0.95.2 (64) and MAXQDA 2022 (65) for the quantitative and qualitative analysis, respectively. The semantic similarity between the model outputs was evaluated using Python 3.10.12 and sentence-transformers 5.0.0. We performed the Friedman test (66) to assess whether there was a significant difference in the reasoning models' conceptual reasoning ability, our primary outcome measure. In the case of a significant result at a significance level of 0.05, pairwise post-hoc comparisons were conducted using Holm-adjusted Wilcoxon signed-rank tests. Missing values were not imputed but still included in the analysis (67, 68).

## 3 Results

In the following, we present our quantitative results (Sec. 3.1) and qualitative results (Sec. 3.2). In summary, the reasoning models were sufficiently reliable and competent or proficient in their clinical reasoning, with no significant or relevant differences between them in terms of conceptual reasoning, completeness, correctness, relevance, or usefulness. However, expert clinicians identified weaknesses in logical coherence, patient-centeredness, empathy, and intuition, with most deviations from expert reasoning in the domain of intuition.

### 3.1 Quantitative results

We observed missing rating values in the domains of conceptual reasoning and usefulness for the initial diagnostic process (20% and 4%), final diagnostic process (20% and 3%), and therapeutic process (20% and 7%). In the therapeutic process, 3% of the completeness, correctness, and relevance scores were also missing.

#### 3.1.1 Initial diagnostic process

**Figure 1:**
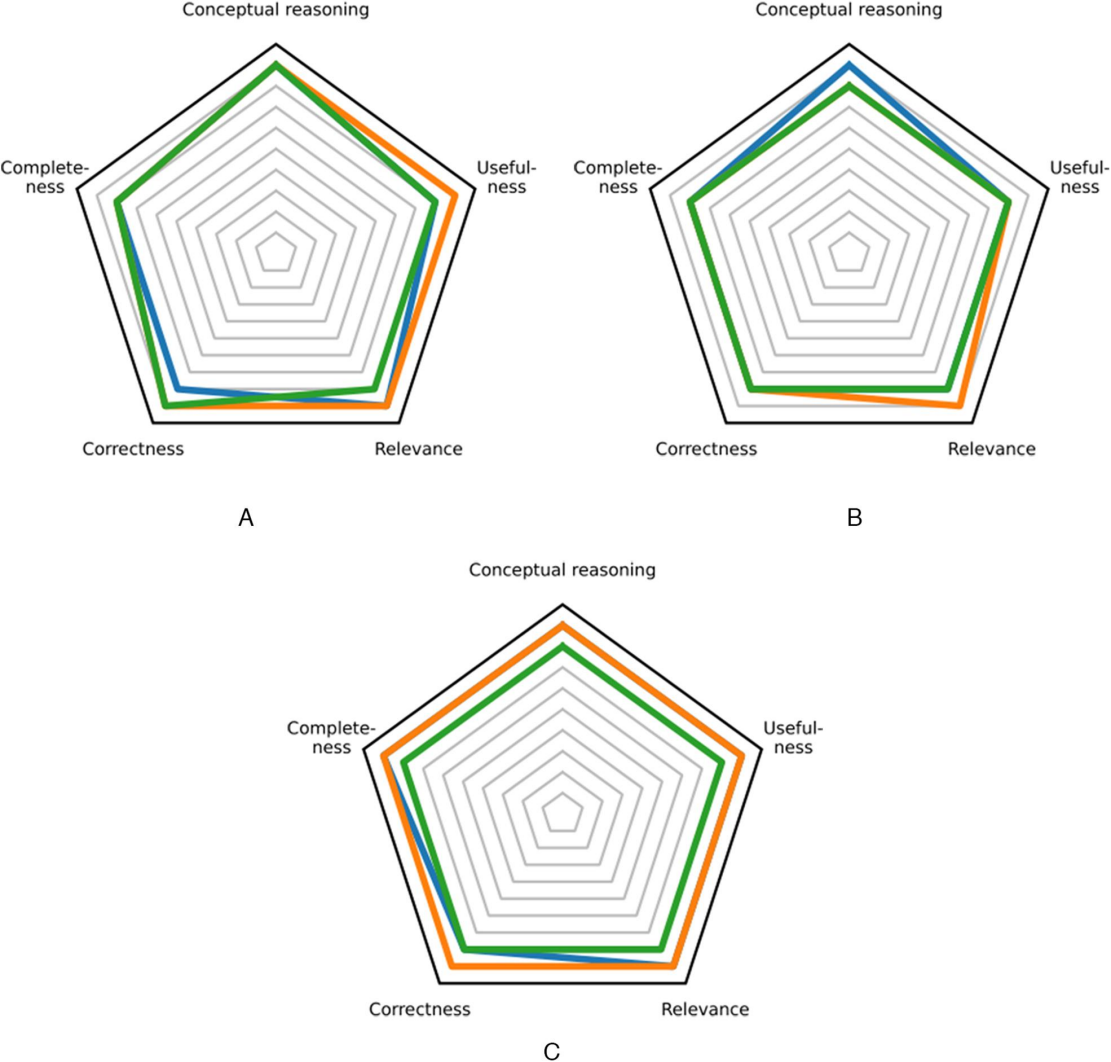
Median performance across reasoning tasks. The results for Gemini 2.5 Pro, o3, and DeepSeek-R1 are shown in blue, orange, and green, respectively. **(A)** Initial diagnostic process. **(B)** Final diagnostic process. **(C)** Therapeutic process.

The median conceptual reasoning rating was 9 (“proficient”) for Gemini 2.5 Pro, o3, and DeepSeek-R1, see Figure 1A. Therefore, there was no statistically significant difference with the Friedman test (*p* ≥ 0.05). Our secondary outcome measures showed median ratings between 8 (“competent”) and 9 (“proficient”) for all reasoning models (Figure 1A). The median completeness score was 8 for Gemini 2.5 Pro, o3, and DeepSeek-R1. For correctness, median scores were 8 for Gemini 2.5 Pro and 9 for o3 and DeepSeek-R1. The median relevance score was 9 for Gemini 2.5 Pro and o3 and 8 for DeepSeek-R1. For usefulness, median scores were 8 for Gemini 2.5 Pro and DeepSeek-R1 and 9 for o3. No model was rated worse than 4 (“intermediate”). Please refer to Supplementary Table 3 for extended results with measures of spread, i.e., the interquartile range (IQR) and range.

#### 3.1.2 Final diagnostic process

The median conceptual reasoning rating was 9 (“proficient”) for Gemini 2.5 Pro and 8 (“competent”) for o3 and DeepSeek-R1, see Figure 1B. The Friedman test showed no statistically significant difference (*p* ≥ 0.05). Our secondary outcome measures yielded median ratings of 8 (“competent”) and 9 (“proficient”) for all reasoning models (Figure 1B). The median completeness score was 8 for Gemini 2.5 Pro, o3, and DeepSeek-R1. For correctness, median scores were also 8 for Gemini 2.5 Pro, o3, and DeepSeek-R1. The median relevance score was 8 for Gemini 2.5 Pro and DeepSeek-R1 and 9 for o3. For usefulness, median scores were 8 for Gemini 2.5 Pro, o3, and DeepSeek-R1. No model was rated worse than 4 (“intermediate”). Please refer to Supplementary Table 4 for extended results with measures of spread.

#### 3.1.3 Therapeutic process

The median conceptual reasoning rating was 9 (“proficient”) for Gemini 2.5 Pro and o3 and 8 (“competent”) for DeepSeek-R1, see Figure 1C. The Friedman test indicated a non-significant trend toward differences between the models [*χ*^2^(*df* = 2, *N* = 20) = 5.82, *p* = 0.054, Kendall's *W* = 0.146]. Our secondary outcome measures again showed median ratings of 8 (“competent”) and 9 (“proficient”) for all reasoning models (Figure 1C). The median completeness score was 9 for Gemini 2.5 Pro and o3, and 8 for DeepSeek-R1. For correctness, median scores were 8 for Gemini 2.5 Pro and DeepSeek-R1 and 9 for o3. The median relevance score was 9 for Gemini 2.5 Pro and o3 and 8 for DeepSeek-R1. For usefulness, median scores were 9 for Gemini 2.5 Pro and o3 and 8 for DeepSeek-R1. No model was rated worse than 5 (“intermediate”). Please refer to Supplementary Table 5 for extended results with measures of spread.

#### 3.1.4 Interrater reliability

The overall interrater reliability was poor, with an ICC(3,5) of 0.12, indicating substantial variability between raters in their evaluations of the reasoning model outputs. When analyzed by outcome measure, the interrater reliability was highest for relevance [ICC(3,5) = 0.30] and correctness [ICC(3,5) = 0.24], followed by completeness [ICC(3,5) = 0.17] and usefulness [ICC(3,5) = 0.09]. Despite providing the most explicit rating instructions (Supplementary Material), there was no agreement between raters for conceptual reasoning [ICC(3,5) = 0.00]. These findings suggest that raters struggled to apply consistent judgment criteria, indicating substantial reasoning heterogeneity and practice variation. This variability may reflect the broader lack of consensus among physiotherapists regarding the management of low-back pain, as well as differences in educational background or clinical training conditions (69–71).

#### 3.1.5 Test-retest reliability

Gemini 2.5 Pro, o3, and DeepSeek-R1 reached median cosine similarities of 0.94, 0.90, and 0.92 across tasks, respectively. For the initial diagnostic process, the median similarities were 0.94, 0.92, and 0.92 for Gemini 2.5 Pro, o3, and DeepSeek-R1, respectively. For the final diagnostic process and the therapeutic process, the median similarities were 0.95, 0.89, and 0.92, as well as 0.93, 0.90, and 0.88, respectively. No model was worse than 0.80. Please refer to Supplementary Table 6 for extended results with measures of spread. Furthermore, Supplementary Table 7 shows that our embedding model appears to produce isotropic text representations. These results suggest that all reasoning models were sufficiently reliable in their outputs, with Gemini 2.5 Pro producing the most consistent answers (at a temperature setting of zero).

### 3.2 Qualitative results

Four subcategories emerged from our qualitative analysis:

1. Logical coherence: This subcategory describes how well the reasoning steps connect to each other and to the conclusion.
2. Patient-centeredness: This dimension captures the consideration of contextual factors, such as individual values, goals, treatment barriers, or psychosocial factors.
3. Intuition: This subcategory reflects intuitive components of fast System 1 thinking (72) like the experience-based pattern recognition that underlies clinical expertise (73)—or the clinical “gut feeling”.
4. Empathy: This dimension expresses the emotional attunement to the patient.

Less frequently, raters expressed feedback in other domains. This included the models' up-to-dateness, such as their overreliance on segment-specific manual therapy (74, 75) or muscle-specific exercise for local stabilizers (4, 76), as well as their overconfidence when faced with clinical ambiguity and uncertainty (77, 78). To structure our qualitative results, though, the following analysis focuses on the strengths, weaknesses, and differences to expert reasoning along the four primary subcategories that emerged from the response material. Table 1 provides an overview of our main qualitative results.

**Table 1:** Main qualitative results across case vignettes, tasks, and model output raters.

Subcategory	Model	Strengths	Weaknesses	Differences to expert reasoning
Logical coherence	Gemini 2.5 Pro	Clear structure; multiple hypotheses; comprehensive, phased treatment plan	Checklist-like; contradictory reasoning; overemphasis on initial hypotheses	Insufficient red flag prioritization; strong biomedical tone that neglected alternative explanations
	o3	Structured, stepwise assessment; organized into domains; concise reasoning	Sequence of tests without meaningful links; premature conclusions	Lacked narrative linking of observations, hypotheses, and decisions
	DeepSeek-R1	Step-by-step process; confirmation/disconfirmation of hypotheses; broad differential diagnosis	Random or poorly prioritized sequences; ambiguous reasoning	Premature working hypothesis; overly textbook-like and rigid; insufficient justification for segmental diagnoses
Patient-centeredness	Gemini 2.5 Pro	Incorporated psychosocial factors; integrated functional limitations and lifestyle factors	Predominantly biomedical; limited exploration of fears and expectations; no shared decision-making	Did not integrate lived experience, values, and context sufficiently
	o3	Addressed beliefs; incorporated psychosocial and lifestyle dimensions; SMART goals and graded exposure	Data-driven; insufficient psychosocial coverage; limited shared decision-making	Felt like research protocols; patient values and life context not central
	DeepSeek-R1	Linked tests and therapy to patient goals; return-to-work focus	Psychosocial factors largely ignored; rigid timelines; limited patient involvement	Approached patient as diagnostic target rather than person with agency
Empathy	Gemini 2.5 Pro	Calm, non-threatening language; reassuring communication in referral situations	Procedural and emotionally distant; no exploration of emotional state	Therapeutic relationship and emotional engagement absent
	o3	/	Detached; no engagement with subjective experience; no reassurance	Therapeutic relationship absent
	DeepSeek-R1	/	Procedural rather than relational; no recognition of emotional impact	Patient “missing” in relational dimension
Intuition	Gemini 2.5 Pro	/	Non-prioritized assessments; rigid commitment (such as sacroiliac joint, motor control impairments)	Lacked “gut feeling”; limited flexible hypothesis weighting
	o3	/	Guideline-based; counterintuitive ordering; merged contributors under single labels	Rigid, data-driven reasoning; lacked moment-to-moment adjustment
	DeepSeek-R1	Limited hypothesis ranking	Over-analyzed details; disproportionate weight to sacroiliac joint and central sensitization	Lacked flexible, experience-based intuition and dynamic hypothesis testing

**Figure 2:**
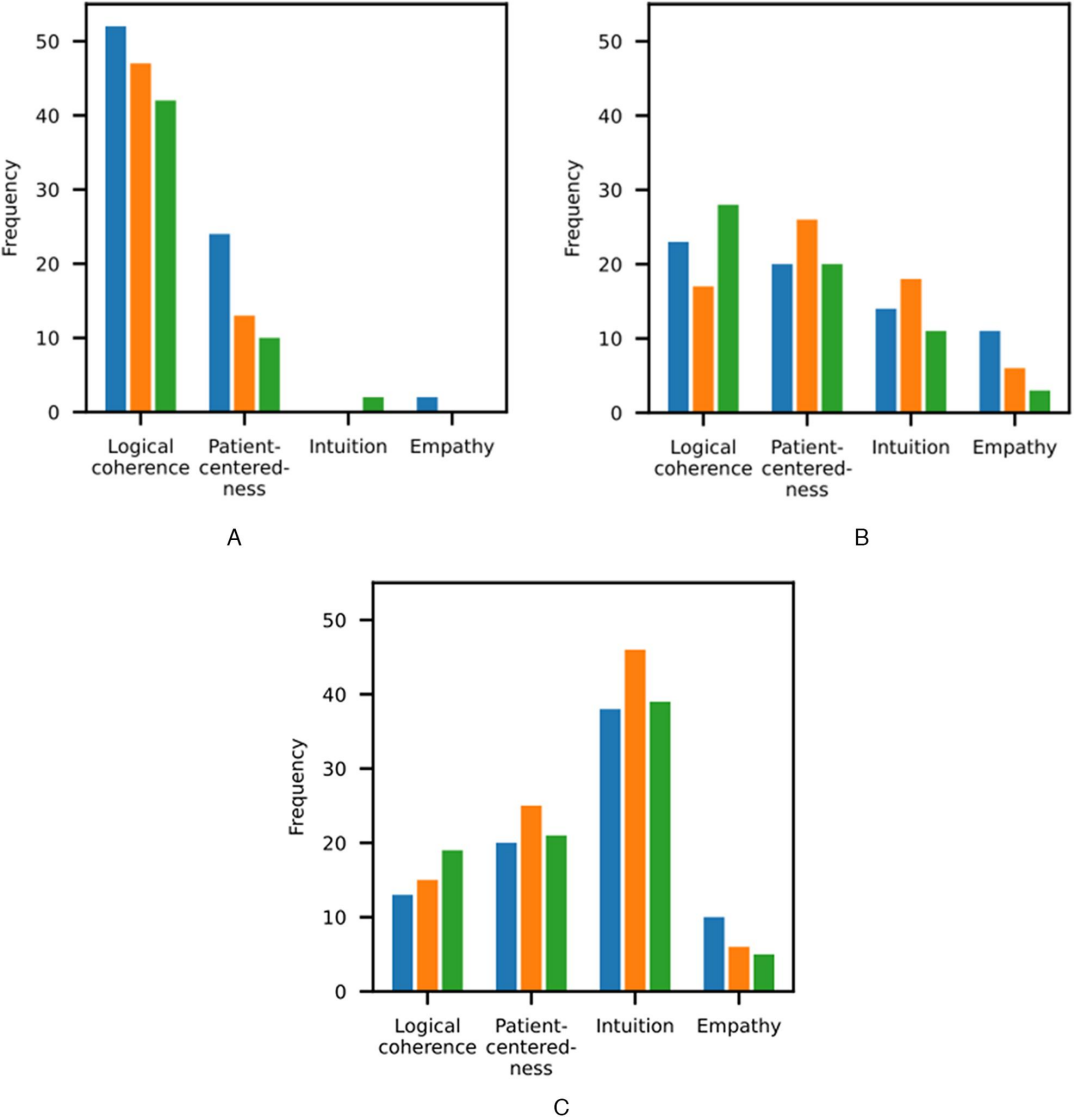
Subcategory frequencies across response categories. The results for Gemini 2.5 Pro, o3, and DeepSeek-R1 are shown in blue, orange, and green, respectively. **(A)** Strengths. **(B)** Weaknesses. **(C)** Differences.

We found that the reasoning models exhibited weaknesses across all subcategories, with most deviations from expert reasoning in the domain of intuition (Figure 2). In the following sections, we provide detailed results for the initial diagnostic process (Sec. 3.2.1), the final diagnostic process (Sec. 3.2.2), and the therapeutic process (Sec. 3.2.3).

#### 3.2.1 Initial diagnostic process

“The AI's reasoning is systematic, comprehensive, and follows guidelines very closely, but feels more ‘checklist-like'.” (Rater 5)

##### 3.2.1.1 Strengths

###### 3.2.1.1.1 Logical coherence

Gemini 2.5 Pro was consistently praised for the clear structure, scope, and clarity of statements. Raters valued its generation of multiple hypotheses, justifications for their inclusion and exclusion, the use of assessments to confirm or refute them, the solid neurological examination, and that the information was presented in clear bullet points. The reasoning was described as transparent with a moderate number of explanations. o3's outputs were also perceived as logically coherent. It followed a structured, stepwise assessment strategy from ruling out serious pathology to pain mechanisms, psychosocial and lifestyle factors, and motor control. DeepSeek-R1 also scored highly on logical coherence, offering a clear step-by-step process, comprehensive confirmation and disconfirmation of hypotheses, appropriate subjective and objective tests, and explicit indications of what to avoid. Its reasoning was described as structured, comprehensive, and well justified, with clear mappings from tests to hypotheses, broad differential diagnosis, and often sensible prioritization of tests in relation to clinical presentation and urgency.

###### 3.2.1.1.2 Patient-centeredness

Gemini 2.5 Pro recognized psychosocial factors and their implications for outcome measure selection. Its clinical reasoning process was described as elaborate in patient-specific characteristics. Raters mentioned that clear primary hypotheses that combine myofascial, motor control, and psychosocial components reflected an attempt to align the diagnostic process with the patients' situations. o3 explicitly addressed the patients' beliefs (for example, by discouraging unnecessary repeated imaging) and systematically incorporated psychosocial and lifestyle dimensions. DeepSeek-R1 showed patient-centeredness by suggesting an expansion of the subjective interview to explore symptom intensity and behavior more deeply, and by linking tests and psychological screening tools to the patients' specific goals, ensuring that reasoning and measurement connected directly to what matters to the patients.

###### 3.2.1.1.3 Empathy

None of the three models received positive codes for empathy. While elements such as conversational tone, moderate number of explanations, or attention to individual goals and beliefs can be interpreted as indirectly supportive of an empathic stance, empathy did not emerge as a distinct strength.

###### 3.2.1.1.4 Intuition

Gemini 2.5 Pro was not explicitly described as intuitive, even though its generation of multiple hypotheses and pattern recognition may superficially resemble intuitive reasoning. For o3, intuition was also not mentioned as a strength. However, DeepSeek-R1 showed limited but noteworthy intuitive qualities. It asked further questions about symptom behavior and provided arguments for ranking hypotheses from most to least probable, suggesting a form of reflective, self-critical reasoning that approximates early stages of clinical intuition.

##### 3.2.1.2 Weaknesses

###### 3.2.1.2.1 Logical coherence

Raters described Gemini 2.5 Pro as sometimes relying on poorly defined terminology and presenting contradictory or poorly justified reasoning. It often used disconnected test sequences and tended to overemphasize initial hypotheses without sufficiently integrating new or conflicting information. o3 was seen as producing checklist-like sequences of tests without a coherent narrative, at times drawing overly specific conclusions or prematurely integrating findings without adequate evidence. DeepSeek-R1, while often structured, was also criticized for random or poorly prioritized assessment sequences, ambiguous reasoning, and inconsistent or inaccurate interpretations.

###### 3.2.1.2.2 Patient-centeredness

Raters noted that Gemini 2.5 Pro's reasoning was predominantly biomedical. It failed to explore the patients' fears and expectations and tended to generate elaborate plans “for” the patients instead of “with” them. o3's assessment and reasoning were also biomedically focused, overlooked important practical and psychosocial aspects, and lacked connection to the person behind the symptoms. Similarly, DeepSeek-R1 did not sufficiently address psychosocial factors. Across all models, raters emphasized a lack of meaningful engagement with the patients' perspectives, goals, and contexts.

###### 3.2.1.2.3 Empathy

Both Gemini 2.5 Pro and o3 avoided follow-up questioning and did not explore important subjective details. As a result, they missed key elements of the therapeutic relationship. DeepSeek-R1 was also unable to understand the patients' emotional state. Overall, the models' communication styles tended to be technical, detached, and lacking in the emotional responsiveness characteristic of human clinical interaction.

###### 3.2.1.2.4 Intuition

Gemini 2.5 Pro did not demonstrate clinical intuition and frequently produced extensive, non-prioritized assessments while failing to consider alternative hypotheses. o3 relied heavily on guidelines, lacked real-time adaptability, and often prematurely confirmed initial assumptions. DeepSeek-R1 demonstrated limited intuitive reasoning. It sometimes over-analyzed details and used disconnected or unnecessary tests. Across models, the absence of contextual sensitivity and experience-based “gut feeling” limited the depth and flexibility of their diagnostic reasoning.

##### 3.2.1.3 Differences to expert reasoning

###### 3.2.1.3.1 Logical coherence

Raters noted that Gemini 2.5 Pro, o3, and DeepSeek-R1 all showed a tendency toward overly systematic, checklist-like reasoning that lacked the integrative, contextualized flow characteristic of musculoskeletal practice. For Gemini 2.5 Pro, they pointed out insufficient red flag prioritization, questionable test selection, and a strong biomedical tone that neglected alternative explanations. o3 was criticized for presenting a sequence of tests without meaningful links or follow-ups. Alternative hypotheses were mentioned but not pursued. DeepSeek-R1's reasoning was described as premature, forming a specific working hypothesis too early in the process. Tests were presented in sequence without clear connections, precise terminology, or explicit red flag integration. The consideration of the patients' tolerance and response to testing was missing.

###### 3.2.1.3.2 Patient-centeredness

Gemini 2.5 Pro's reasoning was frequently described as a sequence of tests without considering the patient's lived experience, values, preferences, or context. o3 was repeatedly criticized for presenting processes that felt like research protocols rather than clinical interactions, offering generic or impractical plans and overlooking psychosocial status or patient-reported outcome measures. It failed to integrate functional relevance or shared decision-making. DeepSeek-R1 also lacked psychosocial assessment, ignored patient load tolerance, and offered extensive test lists that did not adapt to the individuals' capacity or needs. Raters highlighted that their reasoning always incorporates the patients' goals, coping strategies, and resources, which were largely absent from all three models.

###### 3.2.1.3.3 Empathy

For Gemini 2.5 Pro, raters stressed the absence of any engagement with how the patient feels, what worries them, or how their personal circumstances influence their condition. o3 was perceived as similarly detached, missing emotional cues, failing to provide reassurance, and offering no therapeutic relationship. Although DeepSeek-R1 had fewer direct comments on empathy, it was clear the model could not simulate human emotional understanding. Across models, raters emphasized that empathy is not optional. Understanding emotional impact, offering reassurance, and building rapport are integral parts of musculoskeletal practice.

###### 3.2.1.3.4 Intuition

Intuition revealed the sharpest contrast to expert reasoning. Raters repeatedly noted that Gemini 2.5 Pro lacked the ability to read between the lines, did not ask essential follow-up questions, and often missed subtle contextual cues. o3 was viewed as highly data-driven, unable to prioritize hypotheses based on clinical sense, and prone to over-interpreting single tests without waiting for pattern convergence. DeepSeek-R1 showed similar issues, producing highly structured yet rigid reasoning, with raters emphasizing that musculoskeletal practice demands real-time adaptation, flexible hypothesis weighting, and the “gut feeling” formed from experience. Across all models, raters stressed that intuition guides prioritization, hypothesis ranking, test sequencing, and recognition of subtle but meaningful patterns that none of the models could replicate.

#### 3.2.2 Final diagnostic process

“The reasoning was concise and well structured” (Rater 5), but “[not] based on practical experience, observation, interaction, and a biopsychosocial model.” (Rater 3)

##### 3.2.2.1 Strengths

###### 3.2.2.1.1 Logical coherence

Gemini 2.5 Pro was praised for its completeness, clear statements, and explicit instructions, both on what should be done and what should be avoided. Raters highlighted its ability to integrate new assessment findings into an evolving hypothesis and its clear, structured reasoning process that brought together biomechanical, pathoanatomical, and psychosocial factors. o3 also demonstrated solid logical coherence through concise and well-structured reasoning, maintaining openness to alternative hypotheses, and integrating new information meaningfully into an updated working hypothesis. Raters appreciated how o3 organized information into domains—history, pain pattern, palpation, movement, functional findings, lifestyle, and red flags—creating a balanced and systematic diagnostic narrative. DeepSeek-R1 likewise showed strong logical organization, with clear step-by-step explanations, a well-developed integration of subjective and objective findings, and a coherent working hypothesis grounded in differential diagnostics.

###### 3.2.2.1.2 Patient-centeredness

Gemini 2.5 Pro's biopsychosocial orientation was repeatedly described as patient-centered, especially in how it incorporated psychosocial factors, functional limitations, sedentary work behavior, and smoking habits. Raters noted that Gemini 2.5 Pro offered a prognosis that aligned closely with the patient's situation. o3 demonstrated patient-centeredness by integrating signs and symptoms, functional limitations, lifestyle factors, and psychosocial risks into a cohesive picture. Multiple aspects of the patient's lived experience were considered. DeepSeek-R1 was seen as patient-centered due to its incorporation of the patient's own statements, its exploration of contributing factors in a nuanced way, and its clear distinction between primary and secondary symptom drivers. Raters valued how DeepSeek-R1 brought together complex signs and symptoms into a reasoning process that reflected the clinical presentation.

###### 3.2.2.1.3 Empathy

Empathy was not explicitly identified as a strength in any of the models. While some elements, such as clear communication strategies, may indirectly support empathic practice, raters did not describe any of the models as expressing emotional understanding, validating patient experiences, or fostering a therapeutic relationship.

###### 3.2.2.1.4 Intuition

Like empathy, intuition was not mentioned as a strength for any of the models. Raters did not attribute any form of intuitive judgement or flexible “gut-feeling” integration to Gemini 2.5 Pro, o3, or DeepSeek-R1.

##### 3.2.2.2 Weaknesses

###### 3.2.2.2.1 Logical coherence

Gemini 2.5 Pro was criticized for being overly focused on its initial hypotheses and insufficiently attentive to alternatives. Raters also noted contradictory reasoning (for example, switching from disc herniation to sacroiliac joint pain without referencing earlier hypotheses) and that Gemini 2.5 Pro tended to overinterpret findings (for instance, weakness as causative, percussion signs as decisive). o3 was criticized for sometimes presenting information in grid-like lists that lacked explanatory links. Raters pointed out inconsistent terminology and limited coverage of differentials. The diagnostic plan of DeepSeek-R1 was sometimes described as overly narrow or insufficiently detailed. It tended to mix different clinical entities without clear justification and assign diagnoses such as segmental hypermobility without objective support. At times, it also used non-recommended terms such as “neurogenic pain”.

###### 3.2.2.2.2 Patient-centeredness

Gemini 2.5 Pro was described as lacking depth in its psychosocial assessment. Raters noted that the reasoning did not inquire into the patients' life contexts such as coping strategies or personal reasons behind medical decisions. It offered no shared decision-making. o3 also showed limited patient-centeredness, as raters found insufficient coverage of psychosocial factors and patient-reported outcome measures. The model's approach was seen as data-driven rather than patient-driven, with too little attention to feasibility and the patients' story or goals. DeepSeek-R1 had similar limitations. Its reasoning was described as rather biomechanical, with psychosocial factors largely ignored, which created the impression that the patients themselves were missing from the reasoning.

###### 3.2.2.2.3 Empathy

Gemini 2.5 Pro's reasoning was described as procedural and emotionally distant, offering no recognition of the patients' emotional states, fears, or situational contexts. Raters repeatedly noted that the therapeutic relationship was absent. o3 likewise demonstrated no therapeutic empathy, displaying no engagement with the patients' subjective experiences. DeepSeek-R1 was similarly criticized for reducing the patient to diagnostic tasks, with no acknowledgement of emotional impact, personal values, or the need for reassurance.

###### 3.2.2.2.4 Intuition

Gemini 2.5 Pro's reasoning was described as rigid, non-reflective, and unwilling to entertain differentials even when clinically appropriate. Raters pointed out that Gemini 2.5 Pro tended to over-emphasize sacroiliac joint involvement and misunderstand the diagnostic weight of certain tests, reflecting a lack of nuanced clinical judgement. o3's intuition-related weaknesses included counterintuitive ordering of reasoning steps and a tendency to merge different contributors under a single label (for example, “segmental overload”). Raters noted that o3 mixed pain drivers in ways that did not align with typical clinical intuition. DeepSeek-R1 was similarly criticized for lacking intuitive reasoning. It placed disproportionate weight on sacroiliac joint findings, over-interpreted central sensitization for early-stage presentations, and rarely asked further questions.

##### 3.2.2.3 Differences to expert reasoning

###### 3.2.2.3.1 Logical coherence

Gemini 2.5 Pro's reasoning was described as sometimes logically inconsistent. For instance, raters stressed that musculoskeletal reasoning would more carefully connect findings such as age, body mass index, and symptom behavior before concluding passive lumbar instability. For o3, raters noted that its reasoning lacked the narrative threads that physiotherapists provide. They emphasized that musculoskeletal reasoning involves linking observations, hypotheses, and decisions continuously. DeepSeek-R1's reasoning was considered overly textbook-like and sometimes too absolute, with insufficient justification for segmental diagnoses and too rigid for a commitment to its working hypothesis. Raters stressed that their own reasoning would be more cautious regarding terminology, ensure that each step follows logically from the previous one, and avoid over-localization without solid evidence. Across all three models, raters highlighted that human reasoning actively tests and retests hypotheses, adapts with new information, and avoids conclusions unsupported by the clinical picture.

###### 3.2.2.3.2 Patient-centeredness

Gemini 2.5 Pro was repeatedly described as pathology-oriented and insufficiently attentive to patient perspectives, goals, and unique circumstances. Raters noted that Gemini 2.5 Pro did not ask additional subjective questions and failed to integrate the individual's life situation or personal concerns into its reasoning. o3's approach was described as highly data-driven and insufficiently aligned with musculoskeletal practice. Raters emphasized that musculoskeletal reasoning incorporates patient values, lifestyle, fears, previous experiences, and motivation, none of which appeared central in o3's responses. DeepSeek-R1 also differed markedly from expert reasoning by approaching the patient as a list of diagnostic targets rather than a person with agency. Raters highlighted the absence of shared decision-making, person-specific exploration, and adaptation based on patient tolerance. Across models, raters underlined that patient-centered reasoning integrates personal goals, respects limits, prioritizes meaningful functional outcomes, and uses shared decisions to shape the diagnostic pathway. These elements were largely missing in the generated outputs.

###### 3.2.2.3.3 Empathy

Raters consistently stated that the patient was “missing” in the generated responses. For Gemini 2.5 Pro, the absence of emotional context such as psychological needs was explicitly highlighted, alongside the lack of therapeutic relationship-building. o3 likewise showed no evidence of attunement to feelings, values, confidence levels, or concerns, leading raters to stress that musculoskeletal practice requires emotional engagement and rapport building. DeepSeek-R1's reasoning was similarly described as procedural rather than relational, offering no space for empathy-driven decisions or recognition of the patient's emotional landscape. In contrast, raters emphasized that empathy is central to their practice, shaping how they phrase information, frame uncertainty, and tailor decisions collaboratively.

###### 3.2.2.3.4 Intuition

All models failed to weigh the differential diagnoses with sufficient flexibility. For Gemini 2.5 Pro, raters emphasized that physiotherapists would stay more open to alternative hypotheses, especially in non-specific cases, and would avoid treating a single assessment such as pressure biofeedback as definitive. They criticized Gemini 2.5 Pro's tendency to diagnose motor control impairments without sufficient evidence. It was also noted that fear-avoidance must be interpreted contextually because acute pain naturally brings guarded movement, and that Gemini 2.5 Pro did not reflect this nuance. Raters stressed that their own reasoning would incorporate “gut feeling”, functional movement assessment, and contextual information, none of which were visible in Gemini 2.5 Pro's linear, pathology-oriented reasoning. For o3, raters would prioritize functional tasks and movement responses rather than segmental mobility tests, and they would avoid highly anatomical interpretations in long-standing presentations without clear aggravators. Raters emphasized that o3's rigid, data-driven reasoning did not reflect the fluid, moment-to-moment adjustments therapists make based on observation, interaction, and lived clinical experience. DeepSeek-R1 showed similar limitations. Raters criticized the model prematurely narrowing to one hypothesis and emphasized that human reasoning keeps multiple explanations active and tests them dynamically. Raters also highlighted that human therapists incorporate experience-based intuition, especially around the role of sensitization, pain inhibition, and pain-related behaviors, whereas DeepSeek-R1 tended to overlook these nuances.

#### 3.2.3 Therapeutic process

“[The AI suggested a] comprehensive and structured plan of treatment”, but “[a more] individual plan for the patient would be nice.” (Rater 1)

##### 3.2.3.1 Strengths

###### 3.2.3.1.1 Logical coherence

All models offered clear and well-structured treatment concepts. Gemini 2.5 Pro was repeatedly praised for providing a comprehensive, phased treatment plan with clear progression from pain modulation to strengthening and functional restoration. Its plans included exercise, passive modalities, and education, with detailed rationales and communication strategies. Raters highlighted the way Gemini 2.5 Pro often defined rehabilitation phases pragmatically (for example, by number of appointments or outcomes rather than rigid timeframes), the explicit shift from urgent referral and stopping physiotherapy in red flag situations to longer-term rehabilitation “down the road”, and the strong integration of biopsychosocial reasoning into a coherent therapeutic pathway. o3 was described as logically powerful in its therapeutic planning. It frequently offered guideline-based, multimodal treatment plans with clear outcome targets, concrete decision points, and explicit criteria for progression between phases. Raters valued the “pillars” that guided therapy rather than a fixed recipe, the use of SMART (specific, measurable, achievable, relevant, time-bound) goals and bi-weekly re-evaluation, and the seamless “question → answer → treatment consequence” matrix that tied impairments directly to specific interventions. DeepSeek-R1 likewise showed high logical coherence through a phased, well-structured intervention plan. It combined active modalities, strength and conditioning, and clearly justified adjuncts. Raters emphasized the detailed dosage and intensity suggestions, the explicit progression criteria, and the multifactorial approach that linked red flags, motor control training, graded exposure, and functional rehabilitation into a consistent therapeutic logic.

###### 3.2.3.1.2 Patient-centeredness

Gemini 2.5 Pro's plans were considered patient-centered when they explicitly incorporated the patients' goals, used everyday language, and framed treatment within a therapeutic alliance, especially for patients with kinesiophobia. Raters appreciated how the model tried to bridge the gap between therapy and activities of daily living, and how responses acknowledged the severe emotional impact of a likely serious diagnosis and adjusted post-medical rehabilitation strategies accordingly. o3 demonstrated patient-centeredness by offering flexible “pillars” instead of rigid recipes, allowing adaptation to individual needs, and by integrating psychosocial factors and education messages throughout the plan. Its use of SMART goals, graded exposure to feared activities, and regular checkpoints (pain, range of motion, strength, questionnaires) was considered highly supportive of patient-driven progression and shared decision-making. DeepSeek-R1 was viewed as patient-centered where it tied modalities to patient-specific goals, used detailed verbal cues to support execution, and built a phased structure that explicitly targeted functional outcomes and return-to-work. Raters particularly valued the integration of pacing, graded exposure, and lifestyle modifications into a plan that was clearly oriented toward the patients' long-term quality of life and participation.

###### 3.2.3.1.3 Empathy

Explicit positive comments were limited and appeared mainly in relation to Gemini 2.5 Pro. Raters noted that Gemini 2.5 Pro's use of calm, non-threatening language, the clear but reassuring communication around urgent referral, and its later acknowledgement of the emotional burden of a probable serious diagnosis indicated some sensitivity to the patient's emotional state. The inclusion of detailed communication scripts and referral letters were considered supportive for maintaining trust and reducing anxiety. For o3 and DeepSeek-R1, no explicit empathic strengths were reported.

###### 3.2.3.1.4 Intuition

Intuition was not explicitly identified as a helpful aspect of any model. Raters did not attribute intuitive, experience-based treatment adjustments or flexible “gut-feeling” prioritization to Gemini 2.5 Pro, o3, or DeepSeek-R1.

##### 3.2.3.2 Weaknesses

###### 3.2.3.2.1 Logical coherence

Gemini 2.5 Pro lacked explicit progression or regression criteria in its therapeutic plan and tended to work with generic timelines rather than outcome-based milestones. Additionally, certain sections such as manual therapy were overly detailed and risked dominating the active components of therapy. Similarly, o3 tended to use time-based treatment plans without clear milestones. Some raters described its plans as complex and overly specific for non-specific problems. The treatment strategies from DeepSeek-R1 occasionally contradicted themselves (for example, promoting fear-avoidance reduction while simultaneously encouraging abdominal bracing), included interventions that would be unsafe before full medical clearance, and again mostly relied on time-based rehabilitation phases. Raters also criticized DeepSeek-R1 for insufficient connections between reasoning steps and the absence of clear “if–then” decision logic.

###### 3.2.3.2.2 Patient-centeredness

Raters highlighted a shared difficulty in tailoring therapy to patient needs, preferences, and context. Gemini 2.5 Pro frequently offered generic timelines, introduced home programs only after long delays, and failed to involve the patient in decisions or explore existing resources such as sport participation or gym access. The goal setting was considered overly functional and insufficiently linked to participation or meaning. Several raters felt that psychosocial management was missing, leaving important behavioral and emotional factors unaddressed. o3 faced similar concerns. Its therapeutic plans lacked flexibility and were not sufficiently linked to the patients' activities, daily contexts, and psychosocial backgrounds. Strict timelines and dosages risked ignoring patient fluctuations. Furthermore, specific exercise recommendations, intensities, and repetitions were seen as too strict, especially in the absence of real-time patient feedback. Some raters also noted that o3 did not adequately incorporate shared decision-making. DeepSeek-R1's weaknesses in patient-centeredness included rigid timelines, strict exercise prescriptions, insufficient prioritization, and a frequent lack of involvement of the patient in goal setting. Raters felt that the plans could easily overwhelm a real patient, that psychosocial aspects were insufficiently integrated, and that the strong emphasis on detailed protocols detracted from more flexible, collaborative management.

###### 3.2.3.2.3 Empathy

For Gemini 2.5 Pro, raters criticized the absence of therapeutic empathy, the lack of attention to the patients' emotional status, and the limited flexibility to adjust the plan based on setbacks or individual concerns. o3 similarly showed a lack of empathic communication. The treatment plans appeared to be a sequence of steps and exercise parameters rather than a dialogue with a person, and important relational elements, including sensitivity to patient stress, were missing. DeepSeek-R1 also lacked the interpersonal dimension. Raters repeatedly stated that the model offered lists of interventions without the human capacity for empathic adjustment.

###### 3.2.3.2.4 Intuition

None of the models captured the flexible, experience-based judgement that physiotherapists rely on. Gemini 2.5 Pro often used generic timelines rather than outcome-based progressions. o3's recommendations were also frequently based on rigid treatment structures, including strict dosages and highly detailed early-phase programming that may be unrealistic or aggravating in irritable cases. Raters emphasized that real-world clinical intuition includes focusing on high-yield interventions for feasibility, adjusting based on a patient's presentation, and avoiding overload. These elements were missing from o3's dense suggestions. DeepSeek-R1's plans also relied heavily on time-based progression, lacked prioritization of interventions, and tended to over-emphasize certain concepts (such as central sensitization in the first week). DeepSeek-R1 also missed the flexible, conditional reasoning that physiotherapists apply when a patient needs gentler early-phase care.

##### 3.2.3.3 Differences to expert reasoning

###### 3.2.3.3.1 Logical coherence

The models often differed from expert reasoning through overly linear treatment plans. Gemini 2.5 Pro was criticized for frequently presenting treatment sequences without transitions or clear decision points. o3's high level of detail was perceived as unrealistic and disconnected from individual response patterns. Its structure resembled a research protocol rather than the adaptive logic of physiotherapy sessions. DeepSeek-R1 leaned heavily toward passive modalities and sometimes produced contradictory recommendations.

###### 3.2.3.3.2 Patient-centeredness

Gemini 2.5 Pro frequently delivered plans “for” the patients rather than “with” them, lacking shared decision-making, resource exploration, and adaptation to personal context, such as training background, available time, fatigue, mood, or daily fluctuations. Raters stressed that musculoskeletal practice requires co-creation of goals, prioritizing participation rather than purely functional metrics, and adjusting the session content based on the current state. Similar concerns emerged for o3. Its structured program appeared data-driven rather than patient-driven, often with time-based progressions, fixed dosage prescriptions, and minimal space for patient feedback or preference. DeepSeek-R1 was also described as generic in its timings and insufficiently responsive to patient-specific needs. It missed the iterative, flexible planning that characterizes musculoskeletal practice. Furthermore, it did not sufficiently integrate functional outcomes and was too focused on pathomechanical descriptions, lacking psychosocial considerations. Across all three models, raters highlighted that treatment choices and progression depend on patient presentation and continuous reassessment, and that the collaborative clinical conversation elements were largely absent from the generated plans.

###### 3.2.3.3.3 Empathy

For Gemini 2.5 Pro and o3, raters repeatedly noted that the therapeutic relationship, emotional state, and day-to-day wellbeing of the patient were not considered, and that the models provided treatment instructions without showing sensitivity to pain behavior, anxiety, fear, fatigue, or personal meaning of symptoms. DeepSeek-R1 showed similar limitations. Even though it offered detailed technical planning, raters felt that it treated the patient as a list of impairments, missing the concerns and stress that accompany real-world cases. Raters emphasized that empathy is inseparable from therapeutic reasoning, affecting communication style, reassurance, pacing, goal setting, and the patients' sense of safety and agency. The generated output remained procedural and impersonal, without the adaptive emotional attunement central to musculoskeletal practice.

###### 3.2.3.3.4 Intuition

Differences in therapeutic reasoning arose most clearly in the dimension of intuition. Raters emphasized that musculoskeletal reasoning typically begins with a goal and then selects only what is necessary based on research evidence, experience, patient preference, and day-to-day presentation. In contrast, Gemini 2.5 Pro, o3, and DeepSeek-R1 tended to provide highly structured, rigid therapeutic plans. For Gemini 2.5 Pro, raters noted that some of the suggested interventions (such as neural tensioners) would not be introduced so early. They also felt that Gemini 2.5 Pro's large menus of interventions and manual techniques would need to be trimmed substantially in practice, reducing to three or four high-yield interventions per phase with tighter reassessment loops. o3 showed similar divergences. Raters found its plan overly elaborate and “evidence-heavy”, with detailed dosage progressions that are impossible to determine without observing the patients' actual movements, symptoms, and tolerance. They stressed that their reasoning would be more focused on immediate responses, and that graded progressions are often shaped by moment-to-moment patient responses. For DeepSeek-R1, raters mentioned that they would be more considerate not to overwhelm the patient by prioritizing lighter and simpler early-phase strategies.

## 4 Discussion

We present a comprehensive human evaluation of reasoning models for clinical reasoning, encompassing five musculoskeletal case vignettes, three clinical reasoning tasks, three state-of-the-art reasoning models, and five human experts. Our results indicate that all models were sufficiently reliable and competent or proficient in their clinical reasoning, with no significant or relevant differences between them in terms of conceptual reasoning, completeness, correctness, relevance, or usefulness. However, expert evaluations revealed weaknesses in logical coherence, patient-centeredness, empathy, and intuition, with most deviations from expert reasoning in the domain of intuition. In the following, we contextualize our findings with recent work on reasoning models (Sec. 4.1), assess the feasibility of our pilot (Sec. 4.2), discuss its limitations (Sec. 4.3), outline implications for research (Sec. 4.4), and consider the broader impact of our results (Sec. 4.5).

### 4.1 Related work

The benchmarking of LLMs is an active area of research, with most efforts concentrating on evaluating the answer accuracy using predefined question sets (79–83). However, free-text evaluations require a multidimensional assessment of textual outputs to capture the quality of clinical reasoning processes (55–57). Automated evaluation is still limited in its ability to provide such a comprehensive assessment; therefore, human evaluation remains the gold standard (84, 85). To the best of our knowledge, only one other study has examined reasoning models within this context. Safran und Yaşasın (27) assessed one reasoning model [Gemini 2.5 Pro (42)] and two LLMs [GPT-4o (86) and DeepSeek-V3 (87)] for orthopedic treatment planning. In their study, one physiotherapist developed three fictional musculoskeletal cases [rotator cuff tendinopathy, lumbar disc herniation with radiculopathy, and anterior cruciate ligament (ACL) reconstruction], while another physiotherapist rated the model outputs. They evaluated the model performance using the same set of criteria as our secondary outcome measures. In addition, they examined the models' safety awareness (contraindications, red flags, and risks), which we address separately through a dedicated clinical case involving a neoplastic malignant compression of the cauda equina (40). Overall, DeepSeek-V3 performed best, whereas the clinical reasoning capabilities of GPT-4o and Gemini 2.5 Pro were notably limited in terms of their completeness and usefulness, respectively. They also reported qualitative differences between the models regarding their emphasis on lifestyle modification, depth of patient education, and integration of psychosocial factors, with Gemini 2.5 Pro uniquely addressing psychological readiness in ACL rehabilitation. Our study, preregistered before the submission of Safran and Yaşasın's work (88), contributes additional breadth through a larger set of case vignettes, reasoning tasks, and model output raters, as well as reliability analyses.

Interestingly, our finding that reasoning models diverge from musculoskeletal experts primarily on their use of intuition echoes insights from a recent study on how LLMs reason. In our work, intuition or the clinical “gut feeling” reflect intuitive components of fast System 1 thinking (72) like the experience-based pattern recognition that underlies clinical expertise (73). Prior work indicates that slow, explicit reasoning can impair LLM performance on certain tasks that rely on fast, intuitive pattern recognition, similar to human cognition. In implicit statistical learning tasks, for instance, verbalizing reasoning steps can reduce the performance for both humans (89) and LLMs (90). Likewise, in tasks that contain arbitrary exceptions to the rule, requiring self-explanations can harm human performance (91) as well as LLM performance (90). This suggests that models capable of thinking both fast and slow (72), such as hybrid systems with automated routing between non-thinking and thinking modes (92), may be particularly well suited to supporting clinical decision-making. While hybrid systems may mitigate failures arising from over-thinking, limitations rooted in the absence of real-world, embodied experiences are unlikely to be resolved by routing alone. Addressing these gaps may require more resource-intensive solutions, including continued pre-training or targeted fine-tuning on human reasoning traces, to approximate the pattern recognition underlying clinical intuition (93, 94).

### 4.2 Feasibility

Our pilot study demonstrated feasibility regarding recruitment, retention, resources, and assessment (95). We met our target of recruiting five musculoskeletal experts (88), indicating that the recruitment process was practicable at this scale. None of the model output raters reported exceeding the estimated time commitment of 15 working hours, which suggests that the individual workload was acceptable and supports scaling to larger sample sizes. Using the maximum sample standard deviation (1.68) of paired differences across contrasts, tasks, and metrics, we estimate that detecting 1-point differences at a significance level of 0.05 and a power of 0.80 would require approximately 23 raters (96).

Despite these feasibility indicators, the quantitative and qualitative evaluations revealed a non-negligible amount of missing data (Sec. 3.1). On the one hand, some raters reported that the evaluation sheet did not provide sufficiently clear instructions for assessing the model outputs, although the use of NRS ratings without predefined criteria or descriptive anchors is common practice (4, 97). On the other hand, one rater did not assess the primary outcome measure at all, despite this section containing the most explicit guidance from a validated measurement instrument (49, 50). These findings highlight the need to homogenize the rating criteria and enhance the clarity in future iterations of the evaluation sheet. Given the large volume of documents and information involved, rater fatigue may have also contributed to the incomplete data. A more streamlined and perhaps sequential evaluation process may help mitigate this risk in future studies (98).

### 4.3 Limitations

Although we present a comprehensive human evaluation of reasoning models for clinical reasoning, our results should be interpreted with caution. As a pilot investigation, the scope of our study was necessarily limited. We focused exclusively on musculoskeletal low-back pain, diagnostic and therapeutic reasoning, reasoning models from mid-2025, and ratings from five experts at a single institution. A more extensive evaluation—covering other musculoskeletal conditions, prognostic reasoning, more cases, more models, and more experts from multiple institutions—would strengthen the generalizability of our results. More human resources would also make it possible to compare model performance against a true human baseline rather than competency levels from a validated measurement tool (49, 50), to assess test-retest reliability through expert ratings of repeated model outputs rather than semantic similarity (59, 60), to evaluate dynamic interactions with the models rather than static responses (25, 93, 94), and to prospectively collect real-world cases rather than rely on published case reports (36–40). This would also, by design, exclude any potential data leakage from published sources. Last but not least, our performance assessment was informed by the reasoning model traces. However, generated reasoning steps or self-explanations may not necessarily be faithful, i.e., they may not accurately reflect why and how the models actually reached their conclusions (99–102). As a result, there may be a fundamental disconnect between the models' final decisions and the reasoning they present as justification.

### 4.4 Implications for research

First and foremost, we want to emphasize the importance of adopting a comprehensive approach to evaluating LLM outputs (Sec. 2). On the one hand, focusing on output correctness alone fails to account for the inherent heterogeneity in musculoskeletal clinical reasoning (Sec. 3.1). On the other hand, it overlooks critical performance indicators identified in our qualitative analysis, including logical coherence, patient-centeredness, empathy, and intuition (Sec. 3.2). Future research should therefore move beyond accuracy-centric benchmarks and prioritize the development of assessment strategies that more closely reflect the multidimensionality of real-world clinical reasoning. Evidence from physiotherapy research suggests that targeted calibration and structured preoperational training can significantly improve interrater reliability in such multidimensional assessment contexts (69, 103), highlighting the importance of standardized educational frameworks and consensus-based evaluation criteria in these settings.

In terms of model performance, it is noteworthy that we did not find statistically significant or clinically relevant differences between reasoning models (Sec. 3.1). This indicates that open-source models such as DeepSeek-R1 can be viable alternatives to closed-source models like Gemini 2.5 Pro or o3—an important consideration in settings where data-sharing constraints or privacy concerns limit the use of proprietary tools. Looking ahead, we anticipate substantial gains from models that combine fast, intuitive pattern recognition with slow, explicit reasoning (Sec. 4.1). Such hybrid systems may be uniquely positioned to leverage the complementary strengths of non-thinking and thinking modes, allowing them to rely on pattern recognition when appropriate while still engaging in more deliberate processing when needed (72, 92).

Additionally, we expect AI systems to perform better with more elaborate prompt templates. In particular, the weaknesses observed in terms of patient-centeredness and empathy could likely be mitigated by providing models with richer contextual information or more explicit instructions (104–106). For instance, LLMs could be prompted to place greater emphasis on contextual factors, as well as on patients' values, goals, preferences, perspectives, and available resources (Sec. 3.2). They could also be instructed to attend more closely to patients' feelings and concerns, and to offer affective reassurance in order to foster trust and build rapport (Sec. 3.2). An example prompt could read as follows: “Generate a treatment plan and select and justify therapeutic interventions based on a biopsychosocial framework and evidence-based practice. Consider environmental and personal contextual factors in accordance with the ICF, as well as the patient's values, goals, preferences, perspectives, and available resources. Discuss how the patient's emotions and concerns may influence management and specify appropriate communication strategies to address these factors.” Such a prompt could shift models from providing generic exercise plans toward individualized plans that emphasize valued activities, challenge beliefs about “wrong” movements, and accommodate busy work schedules. Future research should further explore which specific prompt engineering techniques yield the most pronounced and consistent gains across populations, tasks, and models.

### 4.5 Broader impact

Reasoning models hold great promise as decision support systems to assist clinicians in managing complex, multifactorial conditions such as musculoskeletal and low-back pain. By supporting the integration of biopsychosocial information within an evidence-based reasoning framework, these models may, in the future, help to facilitate reflective clinical reasoning and reduce unwarranted practice variation (Sec. 3.1). Therefore, the findings of this study may contribute to the broader scientific discussion about the role of artificial intelligence in supporting clinical reasoning in musculoskeletal care and physiotherapy. Integrating reasoning models into digital decision support tools has the potential to enhance resource efficiency in everyday clinical practice while maintaining professional responsibility with the clinician. Beyond clinical decision support, reasoning models may eventually also serve educational purposes, acting as clinical tutors to foster both cognitive and metacognitive skills and to deepen the understanding of biopsychosocial care (107, 108).

However, current reasoning models exhibit important limitations, including weaknesses in logical coherence, patient-centeredness, empathy, and intuition (Sec. 3.2). These gaps raise concerns about the potential reinforcement of biomedical biases in musculoskeletal care, insufficient attention to individual differences, and an increased risk of automation bias (109, 110). Moreover, these models may generate plausible but clinically inappropriate recommendations, especially in contexts requiring experiential insight and intuitive judgement. *At present, the available evidence does not support routine clinical use of reasoning models in musculoskeletal practice.* These systems should therefore currently not be used for clinical decision support or autonomous decision-making outside of controlled research settings. Together, these considerations underscore the need for careful, multidimensional validation and usability testing prior to any clinical deployment to ensure safe, trustworthy, and ethical clinical use.

## Data Availability

The datasets presented in this article are not readily available because they are derived from published case reports and may be subject to copyright and licensing restrictions. Requests to access the datasets should be directed to Ricardo Knauer, ricardo.knauer@htw-berlin.de.

## References

[1] Durant W. The story of philosophy. New York: Pocket Books (2006). (Pocket library, PL 500). Available online at: https://books.google.at/books?id=PXIg5EOsbIQC

[2] Gill TK Mittinty MM March LM Steinmetz JD Culbreth GT Cross M Global, regional, and national burden of other musculoskeletal disorders, 1990–2020, and projections to 2050: a systematic analysis of the Global Burden of Disease Study 2021. Lancet Rheumatol. (202) 5(11):e670–82. 10.1016/S2665-9913(23)00232-1 37927903 PMC10620749

[3] Ferreira ML De Luca K Haile LM Steinmetz JD Culbreth GT Cross M Global, regional, and national burden of low back pain, 1990–2020, its attributable risk factors, and projections to 2050: a systematic analysis of the Global Burden of Disease Study 2021. Lancet Rheumatol. (2023) 5(6):e316–29. 10.1016/S2665-9913(23)00098-X 37273833 PMC10234592

[4] Vibe Fersum K O’Sullivan P Skouen JS Smith A Kvåle A. Efficacy of classification-based cognitive functional therapy in patients with non-specific chronic low back pain: a randomized controlled trial. Eur J Pain. (2013) 17(6):916–28. 10.1002/j.1532-2149.2012.00252.x 23208945 PMC3796866

[5] Engel GL. The need for a new medical model: a challenge for biomedicine. Science. (1977) 196(4286):129–36. 10.1126/science.847460 847460

[6] Habermann M Strube A Büchel C. How control modulates pain. Trends Cogn Sci [Internet]. (2025) 29(1):60–72. 10.1016/j.tics.2024.09.014 39462693

[7] Hagenaars LHA Bos JM Oostendorp RAB. Over de Kunst van Hulpverlenen: Het Meerdimensionale Belasting-belastbaarheidsmodel: Een Vakfilosofisch Model Voor een Menswaardige Gezondheidszorg. Amersfoort: Nederlands Paramedisch Instituut (2006).

[8] Huhn K Gilliland SJ Black LL Wainwright SF Christensen N. Clinical reasoning in physical therapy: a concept analysis. Phys Ther. (2019) 99(4):440–56. 10.1093/ptj/pzy148 30496522

[9] Lapin B Li Y Davin S Stilphen M Johnson JK Benzel E Comparison of stratification techniques for optimal management of patients with chronic low back pain in spine clinics. Spine J. (2023) 23(9):1334–44. 10.1016/j.spinee.2023.04.017 37149152

[10] Rabey M Smith A Kent P Beales D Slater H O’Sullivan P. Chronic low back pain is highly individualised: patterns of classification across three unidimensional subgrouping analyses. Scand J Pain. (2019) 19(4):743–53. 10.1515/sjpain-2019-0073 31256070

[11] Alhowimel A AlOtaibi M Radford K Coulson N. Psychosocial factors associated with change in pain and disability outcomes in chronic low back pain patients treated by physiotherapist: a systematic review. SAGE Open Med. (2018) 6:2050312118757387. 10.1177/2050312118757387 29449945 PMC5808969

[12] Kent P Haines T O’Sullivan P Smith A Campbell A Schutze R Cognitive functional therapy with or without movement sensor biofeedback versus usual care for chronic, disabling low back pain (RESTORE): a randomised, controlled, three-arm, parallel group, phase 3, clinical trial. Lancet. (2023) 401(10391):1866–77. 10.1016/S0140-6736(23)00441-5 37146623

[13] Riley SP Swanson BT Cleland JA. The why, where, and how clinical reasoning model for the evaluation and treatment of patients with low back pain. Braz J Phys Ther. (2021) 25(4):407–14. 10.1016/j.bjpt.2020.12.001 33371952 PMC8353316

[14] Bourassa M Kolb WH Barrett D Wassinger C. Guideline adherent screening and referral: do third year doctor of physical therapy students identify red and yellow flags within descriptive patient cases? A United States based survey study. J Man Manip Ther. (2023) 31(4):253–60. 10.1080/10669817.2023.2170743 36740949 PMC10324444

[15] Driver C Lovell GP Oprescu F. Psychosocial strategies for physiotherapy: a qualitative examination of physiotherapists' Reported training preferences. Nurs Health Sci. (2021) 23(1):136–47. 10.1111/nhs.12771 32860451

[16] Snook AG Arnadottir SA Forbes R. A survey of patient education practices and perceptions of physiotherapists: a mixed methods study. Physiother Theory Pract. (2023) 39(4):772–84. 10.1080/09593985.2022.2025966 35014932

[17] Sackett DL Rosenberg WMC Gray JAM Haynes RB Richardson WS. Evidence based medicine: what it is and what it isn’t. Br Med J. (1996) 312(7023):71–2. 10.1136/bmj.312.7023.71 8555924 PMC2349778

[18] Sokol K Fackler J Vogt JE. Artificial intelligence should genuinely support clinical reasoning and decision making to bridge the translational gap. Npj Digit Med. (2025) 8(1):345. 40494886 10.1038/s41746-025-01725-9 PMC12152152

[19] Xu H Wang Y Xun Y Shao R Jiao Y. Artificial intelligence for clinical reasoning: the reliability challenge and path to evidence-based practice. QJM Int J Med [Internet]. (2025) 118:802–4. 10.1093/qjmed/hcaf114 PMC12778421 40489895

[20] Chen TC Couldwell MW Singer J Singer A Koduri L Kaminski E Assessing the clinical reasoning of ChatGPT for mechanical thrombectomy in patients with stroke. J NeuroInterventional Surg. (2024) 16(3):253–60. 10.1136/jnis-2023-021163 38184368

[21] Hudon A Phan V Charlin B Wittmer R. Teaching clinical reasoning in health care professions learners using AI-generated script concordance tests: mixed methods formative evaluation. JMIR Form Res. (2025) 9:e76618. 10.2196/76618 41264864 PMC12634011

[22] Mansoor I Abdullah M Rizwan MD Fraz MM. Reasoning with large language models in medicine: a systematic review of techniques, challenges and clinical integration. Health Inf Sci Syst. (2026) 14(1):6. 10.1007/s13755-025-00403-0 41323158 PMC12657685

[23] Brodeur PG Buckley TA Kanjee Z Goh E Ling EB Jain P Superhuman performance of a large language model on the reasoning tasks of a physician. arXiv (2024). Available online at: https://arxiv.org/abs/2412.10849 (Accessed November 5, 2025). 10.1126/science.adz4433 42060751

[24] Esteitieh Y Mandal S Laliotis G. Towards metacognitive clinical reasoning: benchmarking MD-PIE against state-of-the-art LLMs in medical decision-making. medRxiv. (2025). 10.1101/2025.01.28.25321282

[25] Qiu P Wu C Liu S Fan Y Zhao W Chen Z Quantifying the reasoning abilities of LLMs on clinical cases. Nat Commun. (2025) 16(1):9799. 10.1038/s41467-025-64769-1 41198657 PMC12592457

[26] Gürses ÖA Özüdoğru A Tuncay F Kararti C. The role of artificial intelligence large language models in personalized rehabilitation programs for knee osteoarthritis: an observational study. J Med Syst. (2025) 49(1):73. 10.1007/s10916-025-02207-x 40459660 PMC12134017

[27] Safran E Yaşasın Y. AI vs AI: clinical reasoning performance of language models in orthopedic rehabilitation. J Health Sci Med. (2025) 8(5):825–31. 10.32322/jhsm.1743257

[28] Barroga E Matanguihan GJ Furuta A Arima M Tsuchiya S Kawahara C Conducting and writing quantitative and qualitative research. J Korean Med Sci. (2023) 38(37):e291. 10.3346/jkms.2023.38.e291 37724495 PMC10506897

[29] Katz-Buonincontro J. Convergent mixed methods designs. In: How to Mix Methods: A Guide to Sequential, Convergent, and Experimental Research Designs. Washington: American Psychological Association (2024). p. 73–82. Available online at: https://content.apa.org/books/17364-005

[30] Schoonenboom J Johnson RB. How to construct a mixed methods research design. Köln Z Soziol Sozialpsychol. (2017) 69(S2):107–31. 10.1007/s11577-017-0454-1 PMC5602001 28989188

[31] Tovin MM Wormley ME. Systematic development of standards for mixed methods reporting in rehabilitation health sciences research. Phys Ther. (2023) 103(11):pzad084. 10.1093/ptj/pzad084 37672215

[32] Ho EKY Chen L Simic M Ashton-James CE Comachio J Wang DXM Psychological interventions for chronic, non-specific low back pain: systematic review with network meta-analysis. Br Med J. (2022) 376:e067718. 10.1136/bmj-2021-067718 35354560 PMC8965745

[33] Li Y Zou C Guo W Han F Fan T Zang L Global burden of low back pain and its attributable risk factors from 1990 to 2021: a comprehensive analysis from the global burden of disease study 2021. Front Public Health. (2024) 12:1480779. 10.3389/fpubh.2024.1480779 39606072 PMC11598917

[34] Vega-Retuerta N Sánchez-Parente S Segura-Jiménez V. Effectiveness of multidisciplinary approaches including exercise to treat non-specific chronic low back pain: a systematic review and meta-analysis across multiple regions. Prev Med. (2025) 199:108381. 10.1016/j.ypmed.2025.108381 40763906

[35] Zaglauer K Kunsorg A Jakob V Görg L Oehlschlägel A Riedel R Effect of a multimodal pain therapy concept including intensive physiotherapy on the perception of pain and the quality of life of patients with chronic back pain: a prospective observational multicenter study named “RütmuS”. Pain Res Manag. (2025) 2025(1):6693678. 10.1155/prm/6693678 40385090 PMC12085246

[36] Caneiro JP Smith A Rabey M Moseley GL O’Sullivan P. Process of change in pain-related fear: clinical insights from a single case report of persistent back pain managed with cognitive functional therapy. J Orthop Sports Phys Ther. (2017) 47(9):637–51. 10.2519/jospt.2017.7371 28704623

[37] Kaple N Phansopkar P. Comprehensive physiotherapy rehabilitation in a 25-year-old female with nonspecific low back pain: a case report. Cureus. (2024) 6:e60514. 10.7759/cureus.60514 PMC11180490 38883141

[38] Mehendale P Iyenagar M Bhatt GD Kothary K. Telerehabilitation for a non-specific low back pain: a case report. Cureus. (2023) 15:e47854. 10.7759/cureus.47854 38021986 PMC10680045

[39] Zimney K Louw A Puentedura EJ. Use of therapeutic neuroscience education to address psychosocial factors associated with acute low back pain: a case report. Physiother Theory Pract. (2014) 30(3):202–9. 10.3109/09593985.2013.856508 24252071

[40] Cataldi F Brindisino F Angilecchia D Andreani A Giovannico G. Neoplastic malignant cord compression mimicking low back pain: a case report. Physiother Res Int. (2023) 28(1):e1971. 10.1002/pri.1971 36068933

[41] Chiang WL Zheng L Sheng Y Angelopoulos AN Li T Li D Chatbot arena: an open platform for evaluating LLMs by human preference. Proceedings of the 41st International Conference on Machine Learning (ICML’24); Vienna, Austria: JMLR.org (2024).

[42] Google DeepMind, Google AI. Gemini 2.5 Pro Model Card. Mountain View (CA): Google/DeepMind (2025). Available online at: https://modelcards.withgoogle.com/assets/documents/gemini-2.5-pro.pdf

[43] OpenAI. OpenAI o3 and o4 System Card. San Francisco (CA): OpenAI (2025). Available online at: https://cdn.openai.com/pdf/2221c875-02dc-4789-800b-e7758f3722c1/o3-and-o4-mini-system-card.pdf (Accessed April 16, 2025).

[44] Guo D Yang D Zhang H Song J Wang P Zhu Q DeepSeek-R1 incentivizes reasoning in LLMs through reinforcement learning. Nature. (2025) 645(8081):633–8. 10.1038/s41586-025-09422-z 40962978 PMC12443585

[45] DeepSeek-AI. Reasoning model (2025). Available online at: https://api-docs.deepseek.com/guides/reasoning_model

[46] Sellergren A Kazemzadeh S Jaroensri T Kiraly A Traverse M Kohlberger T MedGemma technical report. arXiv (2025). Available online at: https://arxiv.org/abs/2507.05201 (Accessed January 7, 2026).

[47] Nori H Lee YT Zhang S Carignan D Edgar R Fusi N Can generalist foundation models outcompete special-purpose tuning? Case Study in Medicine. *arXiv* (2023). Available online at: https://arxiv.org/abs/2311.16452 (Accessed January 7, 2026).

[48] Eckler U Gödl-Purrer B Hurkmans E Igelsböck E Wiederin C. The Physiotherapist Profile of Competencies. Vienna: Physio Austria (2017). Available online at: https://www.physioaustria.at/sites/default/files/2025-10/Profile%20of%20Competencies%20and%20Learning%20Outcomes.pdf

[49] Furze J Gale JR Black L Cochran TM Jensen GM. Clinical reasoning: development of a grading rubric for student assessment. J Phys Ther Educ. (2015) 29(3):34–45. 10.1097/00001416-201529030-00006

[50] Wolden B Wolden M Furze J McDevitt A. Advancing consistency in education: a reliability analysis of the clinical reasoning assessment tool. J Phys Ther Educ. (2024) 39:152–162. 10.1097/JTE.0000000000000365 39116366

[51] Alghadir A Anwer S Iqbal A Iqbal Z. Test–retest reliability, validity, and minimum detectable change of visual analog, numerical rating, and verbal rating scales for measurement of osteoarthritic knee pain. J Pain Res. (2018) 11:851–6. 10.2147/JPR.S158847 29731662 PMC5927184

[52] Thong ISK Jensen MP Miró J Tan G. The validity of pain intensity measures: what do the NRS, VAS, VRS, and FPS-R measure? Scand J Pain. (2018) 18(1):99–107. 10.1515/sjpain-2018-0012 29794282

[53] Hjermstad MJ Fayers PM Haugen DF Caraceni A Hanks GW Loge JH Studies comparing numerical rating scales, verbal rating scales, and visual analogue scales for assessment of pain intensity in adults: a systematic literature review. J Pain Symptom Manage. (2011) 41(6):1073–93. 10.1016/j.jpainsymman.2010.08.016 21621130

[54] Ye W Hackett S Vandevelde C Twigg S Helliwell PS Coates LC. Comparing the visual analog scale and the numerical rating scale in patient-reported outcomes in psoriatic arthritis. J Rheumatol. (2021) 48(6):836–40. 10.3899/jrheum.200928 33262305

[55] Gallifant J Afshar M Ameen S Aphinyanaphongs Y Chen S Cacciamani G The TRIPOD-LLM reporting guideline for studies using large language models. Nat Med. (2025) 31(1):60–9. 10.1038/s41591-024-03425-5 39779929 PMC12104976

[56] Ho CN Tian T Ayers AT Aaron RE Phillips V Wolf RM Qualitative metrics from the biomedical literature for evaluating large language models in clinical decision-making: a narrative review. BMC Med Inform Decis Mak. (2024) 24(1):357. 10.1186/s12911-024-02757-z 39593074 PMC11590327

[57] Lekadir K Frangi AF Porras AR Glocker B Cintas C Langlotz CP FUTURE-AI: international consensus guideline for trustworthy and deployable artificial intelligence in healthcare. Br Med J. (2025) 388:e081554. 39909534 10.1136/bmj-2024-081554 PMC11795397

[58] Koo TK Li MY. A guideline of selecting and reporting intraclass correlation coefficients for reliability research. J Chiropr Med. (2016) 15(2):155–63. 10.1016/j.jcm.2016.02.012 27330520 PMC4913118

[59] Zhang X Zhang Y Long D Xie W Dai Z Tang J mGTE: generalized long-context text representation and reranking models for multilingual text retrieval. Proceedings of the 2024 Conference on Empirical Methods in Natural Language Processing: Industry Track; Miami, Florida, US: Association for Computational Linguistics (2024). p. 1393–412. Available online at: https://aclanthology.org/2024.emnlp-industry.103 (Accessed November 5, 2025).

[60] Cann TJB Dennes B Coan T O’Neill S Williams HTP. Using semantic similarity to measure the echo of strategic communications. EPJ Data Sci. (2025) 14(1):20. 10.1140/epjds/s13688-025-00538-w

[61] Ethayarajh K. How contextual are contextualized word representations? Comparing the geometry of BERT, ELMo, and GPT-2 embeddings. Proceedings of the 2019 Conference on Empirical Methods in Natural Language Processing and the 9th International Joint Conference on Natural Language Processing (EMNLP-IJCNLP); Hong Kong, China: Association for Computational Linguistics (2019). p. 55–65. Available online at: https://www.aclweb.org/anthology/D19-1006

[62] Kuckartz U. Mixed Methods: Methodologie, Forschungsdesigns und Analyseverfahren. Wiesbaden: Springer Fachmedien Wiesbaden (2014). Available online at: https://link.springer.com/10.1007/978-3-531-93267-5

[63] Denzin NK. The Research Act: A Theoretical Introduction to Sociological Methods. 1st ed. New York: Routledge (2017). Available online at: https://www.taylorfrancis.com/books/9781351475273

[64] JASP Team. JASP (Version 0.95.4) [computer software] (2025). Available online at: https://jasp-stats.org/

[65] VERBI Software. Consult. Sozialforschung GmbH. How to Cite MAXQDA. Berlin: MAXQDA Blog (2019). Available online at: https://www.maxqda.com/blogpost/how-to-cite-maxqda (Accessed March 05, 2026).

[66] Eisinga R Heskes T Pelzer B Te Grotenhuis M. Exact *p*-values for pairwise comparison of Friedman rank sums, with application to comparing classifiers. BMC Bioinform. (2017) 18(1):68. 10.1186/s12859-017-1486-2 PMC5267387 28122501

[67] Chaudhuri S Agiwal V. Strategies for preventing and addressing missing data in research. Curr Med Issues. (2024) 22(3):181–3. 10.4103/cmi.cmi_8_24

[68] Thabane L Ma J Chu R Cheng J Ismaila A Rios LP A tutorial on pilot studies: the what, why and how. BMC Med Res Methodol. (2010) 10(1):1. 10.1186/1471-2288-10-1 20053272 PMC2824145

[69] Bahns C Happe L Thiel C Kopkow C. Physical therapy for patients with low back pain in Germany: a survey of current practice. BMC Musculoskelet Disord. (2021) 22(1):563. 10.1186/s12891-021-04422-2 34147077 PMC8214788

[70] Gittinger FP Lemos M Neumann JL Förster J Dohmen D Berke B Interrater reliability in the assessment of physiotherapy students. BMC Med Educ. (2022) 22(1):186. 10.1186/s12909-022-03231-y 35296313 PMC8928589

[71] Kühn L Rosen D Reiter NL Prill R Choi KE. Appropriateness of exercise therapy delivery in chronic low back pain management: cross-sectional online survey of physiotherapy practice in Germany. BMC Musculoskelet Disord. (2024) 25(1):422. 10.1186/s12891-024-07505-y 38811932 PMC11137918

[72] Kahneman D. Thinking, Fast and Slow. New York: Farrar, Straus and Giroux (2013).

[73] Ericsson KA Hoffman RR Kozbelt A Williams AM. The Cambridge Handbook of Expertise and Expert Performance. Cambridge, UK: Cambridge University Press (2018).

[74] Kerry R Young KJ Evans DW Lee E Georgopoulos V Meakins A A modern way to teach and practice manual therapy. Chiropr Man Ther. (2024) 32(1):17. 10.1186/s12998-024-00537-0 PMC11110311 38773515

[75] Nim CG Downie A O’Neill S Kawchuk GN Perle SM Leboeuf-Yde C. The importance of selecting the correct site to apply spinal manipulation when treating spinal pain: myth or reality? A systematic review. Sci Rep. (2021) 11(1):23415. 10.1038/s41598-021-02882-z 34862434 PMC8642385

[76] Saragiotto BT Maher CG Yamato TP Costa LO Menezes Costa LC Ostelo RW Motor control exercise for chronic non-specific low-back pain. Cochrane back and neck group, editor. Cochrane Database Syst Rev. (2016) 2016(11):1465–858. 10.1002/14651858.CD012004 PMC8761501 26742533

[77] Cruz AF Hardt M Mendler-Dünner C. Evaluating language models as risk scores. Proceedings of the 38th International Conference on Neural Information Processing Systems (NIPS ‘24); Red Hook, NY, USA: Curran Associates Inc. (2024).

[78] Xiong M Hu Z Lu X Y LI Fu J He J Can LLMs express their uncertainty? An empirical evaluation of confidence elicitation in LLMs. The Twelfth International Conference on Learning Representations (2024). Available online at: https://openreview.net/forum?id=gjeQKFxFpZ

[79] Pasquale Minervini APAPG Motzfeldt AG Alex B. Openlifescienceai/open_medical_llm_leaderboard. Hugging face (2024). Available online at: https://huggingface.co/spaces/openlifescienceai/open_medical_llm_leaderboard

[80] Hendrycks D Burns C Basart S Zou A Mazeika M Song D Measuring massive multitask language understanding. International Conference on Learning Representations (2021). Available online at: https://openreview.net/forum?id=d7KBjmI3GmQ

[81] Jin D Pan E Oufattole N Weng WH Fang H Szolovits P. What disease does this patient have? A large-scale open domain question answering dataset from medical exams. Appl Sci. (2021) 11(14):6421. 10.3390/app11146421

[82] Jin Q Dhingra B Liu Z Cohen W Lu X. PubMedQA: a dataset for biomedical research question answering. In: Inui K Jiang J Ng V Wan X, editors. Proceedings of the 2019 Conference on Empirical Methods in Natural Language Processing and the 9th International Joint Conference on Natural Language Processing (EMNLP-IJCNLP); Hong Kong, China: Association for Computational Linguistics (2019). p. 2567–77. Available online at: https://aclanthology.org/D19-1259/

[83] Pal A Umapathi LK Sankarasubbu M. MedMCQA: a large-scale multi-subject multi-choice dataset for medical domain question answering. In: Flores G Chen GH Pollard T Ho JC Naumann T, editors. Proceedings of the Conference on Health, Inference, and Learning; Virtual: PMLR (2022). p. 248–60. Available online at: https://proceedings.mlr.press/v174/pal22a.html

[84] Faggioli G Dietz L Clarke CLA Demartini G Hagen M Hauff C Who determines what is relevant? Humans or AI? Why not both? Commun ACM. (2024) 67(4):31–4. 10.1145/3624730

[85] Tam TYC Sivarajkumar S Kapoor S Stolyar AV Polanska K McCarthy KR A framework for human evaluation of large language models in healthcare derived from literature review. Npj Digit Med. (2024) 7(1):258. 10.1038/s41746-024-01258-7 39333376 PMC11437138

[86] OpenAI. GPT-4o System Card. San Francisco (CA): OpenAI (2024). Available online at: https://cdn.openai.com/gpt-4o-system-card.pdf

[87] DeepSeek AI Liu A Feng B Xue B Wang B Wu B DeepSeek-V3 technical report. arXiv (2024). Available online at: https://arxiv.org/abs/2412.19437 (Accessed November 5, 2025).

[88] Knauer R Kalmring M. Valid or void? A mixed-methods pilot study assessing AI’s Clinical reasoning in physiotherapy. OSF Registries (2025). Available online at: https://osf.io/nrtfy/

[89] Fallshore M Schooler JW. Post-encoding verbalization impairs transfer on artificial grammar tasks. Proceedings of the Annual Meeting of the Cognitive Science Society; Cognitive Science Society (1993). Available online at: https://escholarship.org/uc/item/6d40n0m8

[90] Liu R Geng J Wu AJ Sucholutsky I Lombrozo T Griffiths T. Mind your step (by step): chain-of-thought can reduce performance on tasks where thinking makes humans worse. Posters of the 42nd International Conference on Machine Learning; Vancouver, BC, Canada: PMLR/ICML (2025).

[91] Williams JJ Lombrozo T Rehder B. The hazards of explanation: overgeneralization in the face of exceptions. J Exp Psychol Gen. (2013) 142(4):1006–14. 10.1037/a0030996 23294346

[92] OpenAI. GPT-5 System Card. San Francisco (CA): OpenAI (2025). Available online at: https://cdn.openai.com/gpt-5-system-card.pdf

[93] Saab K Freyberg J Park C Strother T Cheng Y Weng WH Advancing conversational diagnostic AI with multimodal reasoning. arXiv (2025). Available online at: https://arxiv.org/abs/2505.04653 (Accessed January 7, 2026). 10.1038/s41591-026-04371-0 PMC13190284 42135531

[94] Tu T Schaekermann M Palepu A Saab K Freyberg J Tanno R Towards conversational diagnostic artificial intelligence. Nature. (2025) 642(8067):442–50. 10.1038/s41586-025-08866-7 40205050 PMC12158756

[95] Aschbrenner KA Kruse G Gallo JJ Plano Clark VL. Applying mixed methods to pilot feasibility studies to inform intervention trials. Pilot Feasibility Stud. (2022) 8(1):217. 10.1186/s40814-022-01178-x 36163045 PMC9511762

[96] Dupont WD Plummer WD. Power and sample size calculations. Control Clin Trials. (1990) 11(2):116–28. 10.1016/0197-2456(90)90005-M 2161310

[97] Horn KK Jennings S Richardson G Van Vliet D Hefford C Abbott JH. The patient-specific functional scale: psychometrics, clinimetrics, and application as a clinical outcome measure. J Orthop Sports Phys Ther. (2012) 42(1):30–42. 10.2519/jospt.2012.3727 22031594

[98] Fetters MD Curry LA Creswell JW. Achieving integration in mixed methods designs—principles and practices. Health Serv Res. (2013) 48(6pt2):2134–56. 10.1111/1475-6773.12117 24279835 PMC4097839

[99] Arcuschin I Janiak J Krzyzanowski R Rajamanoharan S Nanda N Conmy A. Chain-of-Thought reasoning in the wild is not always faithful. Proceedings of the Workshop on Reasoning and Planning for Large Language Models, ICLR 2025. Virtual: ICLR Workshops (2025). Available online at: https://openreview.net/forum?id=L8094Whth0

[100] Chen Y Benton J Radhakrishnan A Uesato J Denison C Schulman J Reasoning models don't always say what they think. arXiv [Preprint]. (2025). 10.48550/arXiv.2505.05410

[101] Chua J Evans O. Are DeepSeek R1 and other reasoning models more faithful? Proceedings of the Workshop on Foundation Models in the Wild, ICLR 2025. Virtual: ICLR Workshops (2025). Available online at: https://openreview.net/pdf?id=rI38nonvF5

[102] Joglekar M Chen J Wu G Yosinski J Wang J Barak B Training LLMs for Honesty via Confessions. San Francisco (CA): OpenAI (2025). Available online at: https://cdn.openai.com/pdf/6216f8bc-187b-4bbb-8932-ba7c40c5553d/confessions_paper.pdf

[103] Vibe Fersum K O’Sullivan PB Kvåle A Skouen JS. Inter-examiner reliability of a classification system for patients with non-specific low back pain. Man Ther. (2009) 14(5):555–61. 10.1016/j.math.2008.08.003 18838331

[104] Howcroft A Bennett-Weston A Khan A Griffiths J Gay S Howick J. AI Chatbots versus human healthcare professionals: a systematic review and meta-analysis of empathy in patient care. Br Med Bull. (2025) 156(1):ldaf017. 10.1093/bmb/ldaf017 41115171 PMC12536877

[105] Schulhoff S Ilie M Balepur N Kahadze K Liu A Si C The prompt report: a systematic survey of prompt engineering techniques. arXiv (2024). Available online at: https://arxiv.org/abs/2406.06608 (Accessed January 7, 2026).

[106] Tabanli A Demirkiran ND. Comparing ChatGPT 3.5 and 4.0 in low back pain patient education: addressing strengths, limitations, and psychosocial challenges. World Neurosurg. (2025) 196:123755. 10.1016/j.wneu.2025.123755 39952398

[107] OpenAI. Jetzt neu: Lernmodus (ChatGPT Study Mode). OpenAI (2025). Available online at: https://openai.com/de-DE/index/chatgpt-study-mode/

[108] Wang J Fan W. The effect of ChatGPT on students' Learning performance, learning perception, and higher-order thinking: insights from a meta-analysis. Humanit Soc Sci Commun. (2025) 12(1):621. 10.1057/s41599-025-04787-y

[109] Goddard K Roudsari A Wyatt JC. Automation bias: a systematic review of frequency, effect mediators, and mitigators. J Am Med Inform Assoc. (2012) 19(1):121–7. 10.1136/amiajnl-2011-000089 21685142 PMC3240751

[110] Lyell D Coiera E. Automation bias and verification complexity: a systematic review. J Am Med Inform Assoc. (2017) 24(2):423–31. 10.1093/jamia/ocw105 27516495 PMC7651899

