# Supplementary material for: Intelligence without intuition: a mixed-methods pilot study on reasoning models in musculoskeletal physiotherapy for low-back pain
Source: Front Digit Health. 2026 Mar 18;8:1789412. doi: 10.3389/fdgth.2026.1789412 (PMC13038865; doi:10.3389/fdgth.2026.1789412)
Supplement: Supplementary file 1 [file Datasheet1.docx]

Supplementary Material

# Survey instrument

**Rating instructions**

Carefully read the case vignette and evaluate the clinical reasoning capabilities of the AI model based on your professional judgement.

**A) Primary outcome measure**

Please rate the conceptual reasoning of the model output using a numeric rating scale from 0 to 10.

Conceptual reasoning: ☐ 0 ☐ 1 ☐ 2 ☐ 3 ☐ 4 ☐ 5 ☐ 6 ☐ 7 ☐ 8 ☐ 9 ☐ 10

**Rating scale definitions:**

0 to 2 (“Beginner”)

- Justifies choice for a **few**tests and measures/interventions
- Able to identify some patient problems
- Interprets results of selected tests/measures

3 to 5 (“Intermediate”)

- Justifies choice for **most**tests and measures/interventions
- Identifies relevant patient problems
- Generates a working hypothesis and begins to prioritize a patient problem list

6 to 8 (“Competent”)

- Justifies choice for **all**tests and measures/interventions
- Prioritizes problem list and incorporates patient goals into plan of care
- Confirms/disproves working hypothesis and determines alternative hypothesis
- Synthesizes relevant patient data

9 to 10 (“Proficient”)

- Generates a hypothesis
- Understands patient perspective
- Reasoning is a fluid, efficient, seamless process (demonstrates “reflection in action”)

**B) Secondary outcome measures**

Please rate the correctness, completeness, relevance, and usefulness of the model output using numeric rating scales from 0 (“Not at all”) to 10 (“Completely”).

Correctness: ☐ 0 ☐ 1 ☐ 2 ☐ 3 ☐ 4 ☐ 5 ☐ 6 ☐ 7 ☐ 8 ☐ 9 ☐ 10

Completeness: ☐ 0 ☐ 1 ☐ 2 ☐ 3 ☐ 4 ☐ 5 ☐ 6 ☐ 7 ☐ 8 ☐ 9 ☐ 10

Relevance: ☐ 0 ☐ 1 ☐ 2 ☐ 3 ☐ 4 ☐ 5 ☐ 6 ☐ 7 ☐ 8 ☐ 9 ☐ 10

Usefulness: ☐ 0 ☐ 1 ☐ 2 ☐ 3 ☐ 4 ☐ 5 ☐ 6 ☐ 7 ☐ 8 ☐ 9 ☐ 10

**C) Qualitative analysis**

Please answer the following questions.

1. What was particularly helpful about the generated output?

_________________________________________________________________________________

2. Where do you see weaknesses in the reasoning process?

_________________________________________________________________________________

3. How do the generated reasoning steps differ from your own?

_________________________________________________________________________________

# Additional results

Table 2: Raters’ years of clinical experience and professional specialization.

| Rater | Years of clinical experience | Professional specialization |
| --- | --- | --- |
| 1 | 11 | Sports physiotherapy |
| 2 | 9 | Sports physiotherapy |
| 3 | 28 | Manual therapy |
| 4 | 24 | Sports physiotherapy |
| 5 | 19 | Manual therapy and rehabilitation |

Table 3: Performance for the initial diagnostic process in terms of the median, IQR, and range across case vignettes and model output raters.

| Outcome measure | Model | Median | IQR | Range | Min-max |
| --- | --- | --- | --- | --- | --- |
| Conceptual reasoning | Gemini 2.5 Pro | 9.00 | 1.00 | 5.00 | 5.00-10.00 |
|  | o3 | 9.00 | 1.50 | 4.00 | 6.00-10.00 |
|  | DeepSeek-R1 | 9.00 | 2.00 | 5.00 | 5.00-10.00 |
| Completeness | Gemini 2.5 Pro | 8.00 | 0.00 | 4.00 | 6.00-10.00 |
|  | o3 | 8.00 | 2.00 | 6.00 | 4.00-10.00 |
|  | DeepSeek-R1 | 8.00 | 1.00 | 4.00 | 6.00-10.00 |
| Correctness | Gemini 2.5 Pro | 8.00 | 1.00 | 5.00 | 5.00-10.00 |
|  | o3 | 9.00 | 1.00 | 5.00 | 5.00-10.00 |
|  | DeepSeek-R1 | 9.00 | 2.00 | 3.00 | 6.00-9.00 |
| Relevance | Gemini 2.5 Pro | 9.00 | 1.00 | 3.00 | 7.00-10.00 |
|  | o3 | 9.00 | 2.00 | 5.00 | 5.00-10.00 |
|  | DeepSeek-R1 | 8.00 | 1.00 | 5.00 | 5.00-10.00 |
| Usefulness | Gemini 2.5 Pro | 8.00 | 1.00 | 3.00 | 7.00-10.00 |
|  | o3 | 9.00 | 1.00 | 5.00 | 5.00-10.00 |
|  | DeepSeek-R1 | 8.00 | 1.00 | 5.00 | 5.00-10.00 |

Table 4: Performance for the final diagnostic process in terms of the median, IQR, and range across case vignettes and model output raters.

| Outcome measure | Model | Median | IQR | Range | Min-max |
| --- | --- | --- | --- | --- | --- |
| Conceptual reasoning | Gemini 2.5 Pro | 9.00 | 1.00 | 3.00 | 7.00-10.00 |
|  | o3 | 8.00 | 1.00 | 5.00 | 5.00-10.00 |
|  | DeepSeek-R1 | 8.00 | 1.00 | 5.00 | 5.00-10.00 |
| Completeness | Gemini 2.5 Pro | 8.00 | 2.00 | 4.00 | 6.00-10.00 |
|  | o3 | 8.00 | 2.00 | 6.00 | 4.00-10.00 |
|  | DeepSeek-R1 | 8.00 | 2.00 | 5.00 | 5.00-10.00 |
| Correctness | Gemini 2.5 Pro | 8.00 | 2.00 | 5.00 | 5.00-10.00 |
|  | o3 | 8.00 | 2.00 | 5.00 | 5.00-10.00 |
|  | DeepSeek-R1 | 8.00 | 2.00 | 5.00 | 5.00-10.00 |
| Relevance | Gemini 2.5 Pro | 8.00 | 2.00 | 4.00 | 6.00-10.00 |
|  | o3 | 9.00 | 2.00 | 5.00 | 5.00-10.00 |
|  | DeepSeek-R1 | 8.00 | 2.00 | 5.00 | 5.00-10.00 |
| Usefulness | Gemini 2.5 Pro | 8.00 | 2.00 | 4.00 | 6.00-10.00 |
|  | o3 | 8.00 | 1.00 | 6.00 | 4.00-10.00 |
|  | DeepSeek-R1 | 8.00 | 2.00 | 5.00 | 5.00-10.00 |

Table 5: Performance for the therapeutic process in terms of the median, IQR, and range across case vignettes and model output raters.

| Outcome measure | Model | Median | IQR | Range | Min-max |
| --- | --- | --- | --- | --- | --- |
| Conceptual reasoning | Gemini 2.5 Pro | 9.00 | 0.25 | 2.00 | 8.00-10.00 |
|  | o3 | 9.00 | 1.00 | 4.00 | 6.00-10.00 |
|  | DeepSeek-R1 | 8.00 | 2.00 | 5.00 | 5.00-10.00 |
| Completeness | Gemini 2.5 Pro | 9.00 | 1.00 | 4.00 | 6.00-10.00 |
|  | o3 | 9.00 | 3.00 | 5.00 | 5.00-10.00 |
|  | DeepSeek-R1 | 8.00 | 2.00 | 5.00 | 5.00-10.00 |
| Correctness | Gemini 2.5 Pro | 8.00 | 1.50 | 4.00 | 6.00-10.00 |
|  | o3 | 9.00 | 2.00 | 4.00 | 6.00-10.00 |
|  | DeepSeek-R1 | 8.00 | 1.00 | 5.00 | 5.00-10.00 |
| Relevance | Gemini 2.5 Pro | 9.00 | 2.00 | 4.00 | 6.00-10.00 |
|  | o3 | 9.00 | 2.00 | 5.00 | 5.00-10.00 |
|  | DeepSeek-R1 | 8.00 | 2.00 | 5.00 | 5.00-10.00 |
| Usefulness | Gemini 2.5 Pro | 9.00 | 1.50 | 4.00 | 6.00-10.00 |
|  | o3 | 9.00 | 1.25 | 5.00 | 5.00-10.00 |
|  | DeepSeek-R1 | 8.00 | 2.00 | 5.00 | 5.00-10.00 |

Table 6: Test-retest reliability, measured with the cosine similarities across five repetitions, in terms of the median, IQR, and range across case vignettes and repetitions. With all values > 0.70, these results suggest that all reasoning models are sufficiently reliable.

| Reasoning task | Model | Median | IQR | Range | Min-max |
| --- | --- | --- | --- | --- | --- |
| Initial diagnostic | Gemini 2.5 Pro | 0.94 | 0.06 | 0.11 | 0.89-1.00 |
|  | o3 | 0.92 | 0.03 | 0.08 | 0.88-0.96 |
|  | DeepSeek-R1 | 0.92 | 0.03 | 0.12 | 0.84-0.96 |
| Final diagnostic | Gemini 2.5 Pro | 0.95 | 0.06 | 0.10 | 0.90-1.00 |
|  | o3 | 0.89 | 0.05 | 0.10 | 0.84-0.94 |
|  | DeepSeek-R1 | 0.92 | 0.03 | 0.13 | 0.82-0.95 |
| Therapeutic | Gemini 2.5 Pro | 0.93 | 0.04 | 0.15 | 0.85-1.00 |
|  | o3 | 0.90 | 0.03 | 0.14 | 0.80-0.94 |
|  | DeepSeek-R1 | 0.88 | 0.05 | 0.14 | 0.82-0.96 |

Table 7: Isotropy assessment for gte-base-en-v1.5 (54), measured with the cosine similarities across five example sentences. Semantically dissimilar sentences show low similarity scores, while similar sentences show higher scores.

|  |  | 1 | 2 | 3 | 4 | 5 |
| --- | --- | --- | --- | --- | --- | --- |
| 1 She sells seashells on the seashore. |  | 1.00 | 0.18 | 0.19 | 0.24 | 0.14 |
| 2 Acute low-back pain, adequate coping strategies, no psychosocial risk factors. |  | 0.18 | 1.00 | 0.71 | 0.65 | 0.55 |
| 3 Chronic low-back pain, decreased local tissue tolerance to heaving work, no psychosocial risk factors. |  | 0.19 | 0.71 | 1.00 | 0.71 | 0.69 |
| 4 Chronic low-back pain, unhelpful illness beliefs, fear avoidance, hypervigilance, passive coping strategies. |  | 0.24 | 0.65 | 0.71 | 1.00 | 0.73 |
| 5 Chronic low-back pain, great stress and anxiety at work, signs of central sensitization, poor expectation of treatment. |  | 0.14 | 0.55 | 0.69 | 0.73 | 1.00 |
